# Oxysterol-Induced Inflammation in Human Diseases: Strategies for Treatment with Natural Compounds and Synthetic Molecules

**DOI:** 10.3390/molecules30132883

**Published:** 2025-07-07

**Authors:** Fatiha Brahmi, John J. Mackrill, Imen Ghzaiel, Leila Rezig, Rym Benkhalifa, Amira Zarrouk, Pierre Jouanny, Anne Vejux, Gérard Lizard

**Affiliations:** 1 Laboratory of Biomathematics, Biochemistry, Biophysics and Scientometrics, Faculty of Natural and Life Sciences, University of Bejaia, Bejaia 06000, Algeria; 2 Department of Physiology, University College Cork, Western Gateway Building, Western Road, T12 XF62 Cork, Ireland; j.mackrill@ucc.ie; 3 Clermont Auvergne INP, CNRS, Institut Pascal, University Clermont Auvergne, 63001 Clermont-Ferrand, France; imenghzaiel93@gmail.com; 4 LR11ES24, LIP-MB ‘Laboratory of Protein Engineering and Bioactive Molecules’, National Institute of Applied Sciences and Technology, University of Carthage, Tunis 1080, Tunisia; l_rezig@yahoo.fr; 5 High Institute of Food Industries, University of Carthage, 58 Alain Savary Street, El Khadra City, Tunis 1003, Tunisia; 6 Laboratory of Biomolecules Venom and Theranostic Applications, Institut Pasteur de Tunis, University of Tunis El Manar, 13 Place Pasteur, BP74, Tunis 1002, Tunisia; rym.benkhalifa@pasteur.tn; 7 LR12ES05, Lab-NAFS ‘Nutrition—Functional Food & Vascular Health’, Faculty of Medicine, University of Monastir, Monastir 5000, Tunisia; zarroukamira@gmail.com; 8 Laboratory of Biochemistry, Faculty of Medicine, University of Sousse, Sousse 4000, Tunisia; 9 Geriatric Internal Medicine Department (Champmaillot), University Hospital Center, Université de Bourgogne Europe, 21000 Dijon, France; pierre.jouanny@u-bourgogne.fr; 10 Centre des Sciences du Goût et de l’Alimentation, CNRS, INRAE, Institut Agro, Université de Bourgogne, 21000 Dijon, France; anne.vejux@u-bourgogne.fr; 11 PHYNOHA Consulting, 21121 Fontaine-lès-Dijon, France

**Keywords:** oxysterols, inflammatory diseases, natural molecules, nutrients, edible oils, synthetic molecules

## Abstract

Oxysterols can be derived from the diet, physiologically produced via specific enzymes, or are generated by autoxidation. These molecules have physiological properties and can also adversely affect vital organs. Indeed, some of them have pro-oxidant and pro-inflammatory activities and can lead to major pathologies. The present review focuses on oxysterols (7-ketocholesterol, 7β-hydroxycholesterol, 25-hydroxycholesterol, 27-hydroxycholesterol, 5,6α-epoxycholesterol, 5,6β-epoxycholesterol, and cholestane-3β, 5α, 6β-triol) involved either in cholesterol metabolism, age-related diseases (such as cardiovascular, neurodegenerative, and eye diseases, e.g., sarcopenia), and inflammatory diseases (especially Behcet’s disease and bowel and lung diseases (e.g., sarcoidosis, COVID-19)). Metabolic pathways associated with oxysterol-induced inflammation are discussed considering the cytokinic TLR4 pathway, non-cytokinic pathways, and the contribution of Ca^2+^ and K^+^ channels. Therapeutic approaches targeting oxysterol-induced inflammation either by natural or synthetic molecules are also presented.

## 1. Introduction

### 1.1. Oxysterols: Origins and Biogenesis

In humans, there are several sources of oxysterols which result from cholesterol oxidation. These compounds can be formed endogenously in different tissues, or can enter the body from the diet [1]. Oxysterols can be generated either by the auto-oxidation of cholesterol following a free radical attack [2], or by specific enzymes, usually of the cytochrome P450 type [3]. In general, the oxysterols oxidized on the steroid nucleus are generated by auto-oxidation, while those oxidized on the side chain result from enzymatic attack [4].

#### 1.1.1. Dietary Origin of Oxysterols

Cholesterol is an important component of many foods. During industrial processes, cholesterol can be subjected to oxidation, leading to the formation of oxysterols [5]. Foods rich in cholesterol such as eggs, egg powder commonly used in processed foods, clarified butter, dairy products, red meat, ham (bacon), and dried or tinned fish are the richest in the following oxysterols: 7-ketocholesterol (7KC), 7β-hydroxycholesterol (7β-OHC), 5,6 α-epoxycholesterol, and 5,6 β-epoxycholesterol [6,7]. During food storage and preparation, cholesterol can easily undergo oxidation when exposed to high temperatures, oxygen or ozone, or light (ultraviolet exposure) [8]. Dehydrated products are particularly sensitive to oxidation [9]. The oxysterols found mainly in food are those oxidized at C7 (7KC, 7β-OHC) as well as epoxycholesterols (5, 6α/5, 6β-epoxycholesterol), but also 25-hydroxycholesterol (25-OHC) formed both enzymatically and by autoxidation [7,10].

In food products, the level of oxysterols can reach 10 to 100 μM [11]. After ingestion of a meal rich in oxysterols (salami, parmesan), assays showed that plasma concentrations of oxysterols (7KC, 7β-OHC) were increased, mainly in chylomicrons, after 5 h. Similar results were shown in rats after gastric infusion of oxysterols [12]. This shows that intestinal absorption varies according to the type of sterol. Also, in rats, it was shown that 92% of dietary oxysterols were absorbed [13]. The majority of ingested oxysterols are absorbed in the form of esters in the upper intestinal tract and taken up in plasma by chylomicrons. Oxysterols can be found in all types of lipoproteins, but the majority are present in low-density lipoprotein (LDL) [14,15]. Depending on their esterification, oxysterols can be transported in the plasma either by albumin or LDL [16]. Measurements carried out with 7KC, 20 α-hydroxycholesterol (20 α-OHC), and 25-OHC have shown that albumin preferentially transports 25-OHC [16,17].

#### 1.1.2. Enzymatic Formation of Oxysterols

Several oxysterols can be produced physiologically by enzymatic oxidation during cholesterol metabolism. A large proportion of oxysterols are formed in this way. In terms of the major oxysterols, 4β-hydroxycholesterol (4β-OHC), 27-hydroxycholesterol (27-OHC), 24(S)-hydroxycholesterol (24(S)-OHC), 7α-OHC, and 25-OHC are produced, respectively, by CYP3A4, CYP27A1, CYP46A1, CYP7A1, and cholesterol 25-hydroxylase (CH25H) [4]. Other enzymes are responsible for the formation of oxysterols, which are generally detected in trace amounts in biological fluids or tissues. Most of these are intermediates in cholesterol metabolism reactions, some of which probably have very short lifetimes [18].

The enzymes involved in cholesterol oxidation are most often members of the cytochrome P450 superfamily of enzymes, but they may also belong to the hydroxysteroid dehydrogenase (HSD) family [19]. CH25H is the exception and is not a member of either of these enzyme families [10]. Not all of these enzymes are ubiquitously expressed, leading to cell-type-specific oxysterol profiles.

CYP3A4 enables the conversion of cholesterol to 4β-OHC, the major oxysterol found in plasma, but also the conversion of cholesterol to 25-OHC [20]. This cytochrome enzyme is expressed in the endoplasmic reticulum (ER) of the liver. It is also involved in the metabolism of many xenobiotics and is estimated to be capable of metabolizing almost half of the drugs used [4,21].

CYP27A1 or 27-hydroxylase converts cholesterol into 27-OHC and is involved in the ‘acidic’ pathway of bile acid formation. This mitochondrial enzyme is expressed in a wide variety of tissues but mainly in the liver, endothelial cells, and monocytes/macrophages [4,21].

CYP46A1 or 24-hydroxylase converts cholesterol to 24(S)-OHC. It is expressed in the ER, mainly in the neurons of the central nervous system, but also in small quantities in the testes and ovaries. It enables the excretion of cerebral cholesterol, as cholesterol cannot cross the blood–brain barrier (BBB), whereas 24(S)-OHC can [4,21].

CYP7A1 or 7α-hydroxylase is the first enzyme involved in the ‘classic’ bile acid formation pathway. It converts cholesterol into 7α-OHC. It is highly expressed in the liver and more specifically in within the ER of hepatocytes. Its expression is regulated by bile acid levels [21]. It can also produce 7KC from 7-dehydrocholesterol [4].

CH25H or 25-hydroxylase catalyzes the conversion of cholesterol into 25-OHC. It is expressed in the ER of most tissues. It is not a member of the cytochrome enzyme family and is a non-heme iron-containing protein [21,22].

#### 1.1.3. Formation of Oxysterols by Autoxidation

The carbons in rings A and B of the sterane nucleus of cholesterol are the most sensitive to free radical attack, particularly carbons 5, 6, and 7. There are two types of autoxidation, type I and II.

Type I autoxidation involves oxidation by free radicals (superoxide anion (O_2_•^−^), hydrogen peroxide (H_2_O_2_), hydroxyl radical (HO^•^), nitric oxide (NO), and peroxynitrites ONOO^−^). These can be generated by cellular metabolism, or by their subsequent decomposition into hydroxyl radicals, by the dismutation of two superoxide anions into hydrogen peroxide (2 O_2_^•−^ → H_2_O_2_) or by the Fenton reaction (H_2_O_2_ + Me^n+^ → HO^•^ + Me^(n+1)+^) where Me is a transition metal such as copper, iron, or aluminum [8]. Most often, oxidation by ROS or reactive nitrogen species (RNS) result in the loss of a hydrogen on carbon 7, due to the weak bond between carbon and hydrogen. The dissociation energy of this bond is 88 kcal/mol [23]. As this local oxidation at C7 is fairly stable, it can then easily react with molecular oxygen to form a peroxyl radical (COO^•^). Subsequently, by reacting with another hydrogen lost by another molecule, the peroxyl radical will form a cholesterol hydroperoxide (7α- or 7β-OOHC). As the hydroperoxide function is very unstable, hydroperoxide cholesterol breaks down into 7α-OHC, 7β-OHC, or 7KC. These three oxidized C7 oxysterols are the ones formed mainly by auto-oxidation [24]. 7KC is the major oxysterol in OxLDL, accounting for around 30% of total sterols [25,26].

Type II cholesterol auto-oxidation involves non-radical attack by oxygen singletons (1ΔgO_2_, hypoclorous acid (HOCl) or ozone (O_3_)). The involvement of ozone is interesting given that it is linked to atmospheric pollution. Oxygen singletons can be formed by the reaction of hydrogen peroxide (H_2_O_2_) with HOCl, both of which are produced during inflammatory reactions in the presence of myeloperoxidase. According to Iuliano [8] C5, C6, C7 hydroperoxides, 5,6-epoxides (α/β), as well as secosterols, are formed as primary compounds in these reactions [23]. The 5,6α/β-epoxycholesterols can be taken up by the enzyme cholesterol epoxide hydrolase to form cholestane-3β, 5α, 6β-triol. It has been shown that aminolysis of α-epoxycholesterols can give alkylamino-oxysterols. These include dendrogenin A, which can be formed when a 5,6α-epoxycholesterol reacts with a histamine through the action of an enzyme that has yet to be identified at the molecular level, dendrogenin A synthase [27,28,29].

## 2. Involvement of Oxysterols in Inflammatory Human Diseases

**Figure 1:**
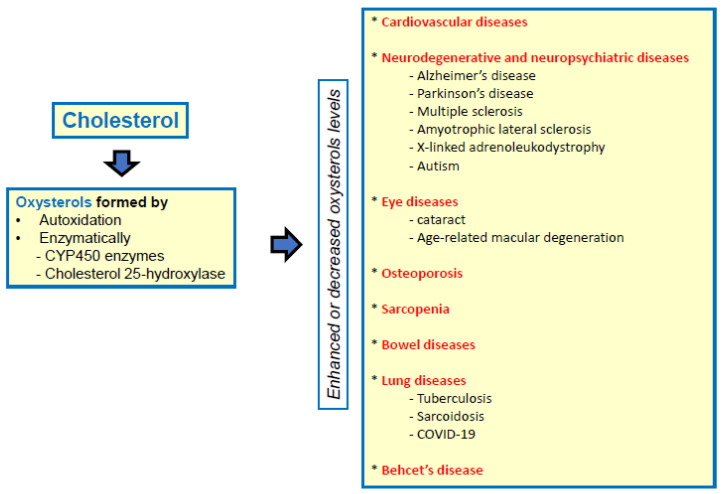
Involvement of cholesterol-oxidized products in inflammatory diseases, with a focus on cardiovascular, neurodegenerative, eye, osteoporosis, sarcopenia, bowel, lung and Behcet’s diseases.

There is substantial evidence that oxysterols can adversely affect certain major and vital organs such as the heart, brain, blood vessels, bones, pancreas, and eyes [30]. These oxysterols might play a role in the development and course of chronic illness [30,31] and also of infectious diseases, especially COVID-19 [32,33] (Figure 1). Actually, a number of clinical studies have shown that people with type 2 diabetes, obesity, hypercholesterolemia, and atherosclerosis have higher levels of various oxysterols [34,35]. These can be used as useful indicators to diagnose certain pathologies, or to forecast the occurrence and progression of diseases such as cardiovascular diseases, Alzheimer’s disease, diabetes mellitus, multiple sclerosis, osteoporosis, lung cancer, breast cancer, and infertility [36]. In addition, some oxysterols could be also used as biomarkers of some rare diseases such as X-linked adrenoleukodystrophy (X-ALD) [37] and Niemann–Pick disease [38,39]. It is noteworthy that oxysterols involved in inflammatory diseases have often simultaneously pro-oxidant and pro-inflammatory properties contributing to the aggravation of symptoms via an overproduction of pro-inflammatory cytokines at the system level or in the tissues affected by the disease [40,41,42].

### 2.1. Cardiovascular Diseases

Cardiovascular disease, often associated with atherosclerosis, is the main cause of morbidity and death. Atherosclerosis is a lipid metabolic issue in addition to a chronic inflammatory illness [43]. Oxysterols are the main component that can aid in the development of atherosclerosis and have been demonstrated in numerous empirical investigations to have a role at different stages of the atherosclerotic process [30,44]. Patients with atherosclerotic lesions or cardiovascular illnesses have higher plasma levels of oxysterols [36]. The oxysterols found mainly in atheromatous plaques are mainly 7KC, 7β-OHC, 7α-OHC, 5,6α-epoxycholesterol, 5, 6β-epoxycholesterol, and cholestane-3β, 5α, 6β-triol; they can reach levels up to 100 times higher than normal plasma levels [45,46]. 7α- and 7β-OHC comprise 75–85% of oxysterols present in the plaques at various locations. They are also among the most important oxysterols that are present in an atherosclerotic lesion. Their amount increases with the degree of atherosclerosis and is almost directly correlated with cholesterol levels [30]. Atherosclerotic patients’ plasma and atheromatous plaques have been found to include certain oxysterols (7β-OHC, 7KC, and 25-OHC). These oxysterols are strong in vitro inducers of MCP-1, MIP-1β, TNF-α, and/or IL-8 secretion, the latter of which involves the MEK/ERK1/2 cell signaling pathway [41].

An increase in 7β-OHC appears to be a biomarker of cardiovascular risk [47]. Björkhem showed that there was a considerable rise in 7β-OHC levels in patients exhibiting rapid progression of carotid atherosclerosis [48].

Due to its pro-oxidant and pro-inflammatory properties, it is now well established that 7KC contributes to the development of atherosclerosis [31]. 7KC has been shown to contribute to the pathophysiology of atherosclerotic plaques. It induces an increase in the expression of cell adhesion molecules (Inter Cellular Adhesion Molecule-1 (ICAM-1), Vascular Cell Adhesion Protein-1 (VCAM-1) and E-selectin due to the elevation of ROS at vascular endothelial cells level, which promotes the recruitment of macrophages to plaques in the early stages of the pathology [26].

A recent study investigated in-depth proteomic profiling and showed the effects of 7KC on the macrophage proteome. Atherogenic/M1 indicators, cholesterol metabolism, biosynthesis and transport, and nutrient transport in general were among the dynamic alterations that 7KC independently mediated. These effects prime the macrophage, increasing the release of important pro-inflammatory factors, including TNFα release triggered by LPS [49].

In human atherosclerotic plaque-derived monocytes and macrophages, 25-OHC contributes to the production of foam cells and stimulates the release of pro-inflammatory cytokines and chemokines, including IL-1, IL-6, IL-8, CCL5, and M-CSF. However, in certain situations, 25-OHC can block the Akt/NFB signaling pathway, induce IFN, repress SREBP, and antagonize inflammasomes, all of which can reduce the activity of inflammasomes [50].

Other oxysterols aid in the rupture and erosion of plaque in advanced atherosclerotic lesions. For instance, it was noted that α-triol promotes smooth muscle cell calcification, which damages the artery wall [51].

### 2.2. Neurodegenerative Diseases

Among the neurodegenerative diseases where the increase of the oxysterols amount was noticed are Alzheimer’s disease, Parkinson’s disease, amyotrophic lateral sclerosis (ALS), Huntington’s disease, multiple sclerosis, and X-linked adrenoleukodystrophy (X-ALD). The most commonly accepted forms of oxysterols that may contribute to the pathophysiology of neurodegenerative disease phases are 24(S)-OHC and 27-OHC, 7KC, 7β-OHC, 7α-OHC, 25-OHC, 27-OHC, 5,6α-epoxycholesterol, 5,6 β-epoxycholesterol, 4β-OHC, and 4α-OHC [52].

Variations in the levels of 24(S)-OHC and 27-OHC in the blood and/or cerebrospinal fluid have been linked to a number of neurological illnesses. To remove extra cholesterol from the brain, CYP46A1 almost exclusively produces 24(S)-OHC in neurons; this oxysterol, unlike cholesterol, can pass through the BBB [53] and is linked to an activation of nicotinamide adenine dinucleotide phosphate oxidase (NADPH-oxidase or NOX), whose activity is itself modulated by oxysterols [54].

7KC influences neurodegeneration by acting on the interactions of the Aβ 1-42 peptide with the plasma membrane, promoting its incorporation into fibrillar deposits [55]. These results on the relationships between 7KC and Aβ 1–42 on synthetic lipid membranes, or cells in culture, are in agreement with in vivo observations of patient brains [56]. In addition, the effects of 7KC on microglia, particularly at the lysosomal level, could lead to a reduction in the efficiency of phagocytosis of the Aβ 1–42 peptide and promote the appearance of amyloid plaques [57]. Izumi, et al. [58] demonstrated the impact of 25-OHC on hippocampal plasticity and learning involve NLRP3 inflammasome and cellular stress responses in multiple neuropsychiatric illnesses.

Apart from these general findings, we will indicate the impact of oxysterols in the most pronounced neurodegenerative diseases separately and in a more detailed manner.

#### 2.2.1. Alzheimer’s Disease

The most prevalent kind of dementia, Alzheimer’s disease (AD), causes cognitive impairment and progressive cognitive loss. Numerous studies, with varying methodologies and findings, have been conducted and are currently underway to examine the significance of circulating oxidized cholesterols in AD [59].

The identification of the apolipoprotein E (ApoE) ε4 allele’s genetic link with both familial and sporadic late-onset AD raised the prospect of lipid metabolism and transport abnormalities in AD patients’ brains [60].

In early-onset Alzheimer’s disease, there is an elevation of 27-OHC in the blood plasma and CSF fluid, which is thought to be a risk factor. In addition to decreasing brain glucose uptake, GLUT4 expression, and spatial memory, the release of 27-OHC from circulation into the brain can also activate the renin–angiotensin system (RAS), which can result in ischemic brain injury, oxidative stress, and compromised cognitive performance. Furthermore, hypertension and insulin resistance, two risk factors for Alzheimer’s disease, may be exacerbated by RAS activation [61].

One of the primary ways that excess cholesterol is eliminated from the brain is by the neuron-specific enzyme CYP46A1 converting it to 24(S)-OHC [62]. When comparing to control in AD, some studies indicated elevated amounts of 24(S)-OHC in the blood [63,64] but others discovered tiny quantities [65,66].

According to An et al. [67] who used random forest machine learning and 5-fold cross-validation, a panel of five oxysterols were identified that could distinguish patients with mild cognitive impairment (MCI) from controls with good performance. So, given their role in controlling neuronal death, neuroinflammation, oxidative stress, Aβ accumulation, and other molecular pathways underlying AD pathology, the intracerebral and extracerebral equilibrium of two side-chain enzymatical oxysterols—specifically, the flux of 24(S)-OHC from the brain into the circulation and the reverse flux of 27-OHC—has been identified as a critical factor in AD pathogenesis. As a result, these oxysterol patterns in biological fluids and organs might be better markers of disease than specific individual components.

In rats, cognitive performance and cholesterol metabolism were adversely affected when 26-OHC was administered via the tail vein [68]. High plasma levels of 26-OHC were found to be strongly associated with moderate cognitive impairment (MCI) [69]. In comparison to controls, Popp, Meichsner, Kölsch, Lewczuk, Maier, Kornhuber, Jessen and Lütjohann [63] and Zarrouk, et al. [70] reported high amounts of 26-OHC in the bloodstream while Mateos, et al. [71] and Hughes, Kuller, Lopez, Becker, Evans, Sutton-Tyrrell and Rosano [64] reported low levels, and Costa, Joaquim, Nunes, Kerr, Ferreira, Forlenza, Gattaz and Talib [65] found no difference. Otherwise, there were reports of elevated levels of 7-oxycholesterol in AD [72].

When 24(S)-OHC and 27-OHC are present, β-amyloidogenesis—which is connected to Alzheimer’s disease—increases and proteins linked to Parkinson’s disease fluctuate in expression [73].

Radhakrishnan, et al. [74] noticed increased 7KC levels in the brains of 3xTg mouse model of AD. Microglia activation and elevated oxidative stress in astrocytes were the outcomes of applying 7KC to a microglia cell line alone or to mixed astrocyte and microglia cultures.

According to Choi, et al. [75], 25-OHC in AD, when administered in vivo, mechanistically increase the esterification of cholesterol, alters the dynamics of cell membranes, further decreases phagocytosis, raises the synthesis of pro-inflammatory cytokines, and hinders microglial surveillance. Furthermore, it has been demonstrated that amyloid-beta (Aβ) increases 25-OHC levels and CH25H expression in microglia, worsening these functional deficits.

Papotti et al. [76] measured and correlated particular lipid markers in patients with varying levels of cognitive decline, such as those with AD and those with MCI related to AD (MCI-AD) carriers or non-carriers of the APOE4 genotype. They concluded that compared to non-carriers, AD APOE4 carriers displayed increased PCSK9 and 24(S)-OHC in CSF. CSF PCSK9 and 27-HOC were negatively correlated in AD, and CSF PCSK9 and 24(S)-OHC were negatively correlated only in AD APOE4 carriers. In AD, APOE4 carriers displayed a positive association between CSF and serum PCSK9, indicating PCSK9 exchange between the brain and the periphery. So, ApoE4-associated lipid changes in AD may be specifically indicated by PCSK9 and 24(S)-OHC, which may aid in the clinical development of the AD spectrum.

A total of 142 people between the ages of 49 and 88 were included in an AD study. Subjects with AD pathology had greater levels of cerebrospinal fluid (CSF) 24S-OHC and a higher 24(S)-OHC/27-OHC ratio. Aβ1–42 levels were associated with CSF desmosterol [77].

Postmortem frozen brain tissue CSF from patients with late-stage AD (Braak stages III–IV) and early-stage AD (Braak stages I–II) were examined for lipids. Brain tissue and mitochondria isolated from late-stage AD brain tissue had increased amounts of oxysterols namely 26-OHC, 25-OHC, and 7-oxycholesterol, with the exception of 24(S)-OHC, which was lower in late AD [78].

#### 2.2.2. Parkinson’s Disease

Recognition of Parkinson disease (PD) is primarily based on clinical findings that link numerous nonmotor characteristics, including hyposmia, sleep disorders, behavioral or mental health issues, and dysautonomia, with complex motor impairment, or Parkinsonism, which includes rigidity, akinesia, rest tremor, and gait disturbance [79]. Oxysterols can control proteins implicated in Parkinson’s disease progression. LXR and estrogen receptors are two types of nuclear receptors that were engaged in the mechanisms of this regulation [80].

By enhancing LXR-mediated transcription of alpha-synuclein and decreasing estrogen receptor-mediated transcription of tyrosine hydroxylase, 27-OHC favorably regulates the expression of this protein [80]. It has also been demonstrated that the LXR pathway, namely 27-OHC, regulates alpha-synuclein expression in SK-N-SH neuroblastoma cells and MO3.13 oligodendrocyte cell lines [81].

The severity of the disease was associated with 24(S)-OHC levels in CSF, and some patients have higher 27-OHC levels in their CSF [48]. Feeding of rabbits with a diet supplemented with 2% cholesterol for 12 weeks revealed that long-term ingestion of this kind of food caused an increase in alpha-synuclein in the substantia nigra of these animals [82].

In Parkinson’s disease, elevated levels of 24(S)-OHC, 27-OHC, 7β-OHC, and 7KC have been measured in the visual cortex [45,83].

#### 2.2.3. Multiple Sclerosis

Immune-mediated demyelination and axon loss in the central nervous system are hallmarks of multiple sclerosis (MS). Based on an array of investigations, oxysterols in MS patients may serve as indicators of particular disease stages [84]. In MS, 24(S)-OHC may be an appropriate biomarker of brain damage and neuronal metabolism [85].

One well-established indicator of neuroaxonal damage in MS is serum neurofilament light chain (sNfL). At follow-up, there was a positive correlation between 7KC and 7β-HOC and sNfL levels. After controlling for LDL-C or HDL-C, the relationships between 7KC or 7β-HOC and sNfL were still significant [86].

Research revealed that individuals with MS experienced a considerable drop in 24(S)-OHC levels [87]. Furthermore, 24(S)-OHC diffuses into the cerebrospinal fluid in cases of neuronal degeneration and may have lipotoxic effects on many central nervous system cells, including oligodendrocytes [88].

Serum 24(S)-OHC levels show a negative correlation with normalized brain volume measurements in patients with MS who have relapses [89]. This discovery also indicates a potential involvement of these oxysterols in MS, since elevated levels of 24(S)-OHC and 27-OHC have been documented in patients with a comparable illness [90]. Patients with MS in the progressive phase also had higher circulating levels of 15-OHC and 15-KC [87].

Higher levels of total hydroxy-octadecanoic acid (total HODEs), including 9-HODE and 12-HODE, were also found in patients with MS. This oxidative stress biomarker (total HODEs) was linked to an elevated level of 7KC and 7β-OHC, which are primarily produced by auto-oxidation, and additionally to an increase in 24(S)-OHC, which may be a sign of neuronal death [91].

During times when the disease is active, patients with MS have higher fluxes of 24(S)-OHC, which can lead to elevated plasma levels of this oxysterol. Furthermore, there is a correlation between the amount of brain atrophy and the plasma levels of this oxysterol in the blood. An elevated flux of 24(S)-OHC from the brain into the cerebrospinal fluid (CSF) in patients who have neuronal injury and/or demyelination was also recorded. Otherwise, increased 27-OHC flow from the circulation into the CSF is caused by a BBB deficiency [92].

#### 2.2.4. Amyotrophic Lateral Sclerosis

Amyotrophic lateral sclerosis (ALS) is an adult-onset non-demyelinating neurodegenerative illness, leading to motor impairments. There are two types: sporadic (90% of cases) and familial (caused by mutations in over 20 genes). Oxysterols may be involved in ALS because of their partial capacity to bind the LXR receptors, as well as other receptors like oxysterol binding proteins (OSBP) [93].

Compared to the control (persons without ALS) and treatment groups (ALS patients treated with riluzole), an untreated ALS group had greater amounts of 24-OHC and 25-OHC in their CSF. 25-OHC may also be directly involved in the pathophysiology of ALS through GSK3-ß activation and neuronal apoptosis [94]. It has been shown that 25-OHC can restore the membrane and functional characteristics of neuromuscular junctions in the early stages of the disease [95]. The CYP27A1 enzyme is shown to be less active in ALS patients, which makes it more difficult for the central nervous system to eliminate excess cholesterol that could be harmful to neuronal cells. This is further exacerbated by a decrease in the neuroprotective LXR ligands such as 3,7-diHCA [96]. On the other hand, in spite of the fact that levels of 24(S)-OHC, 27-OHC, and 25-OHC are typically higher in ALS patients, there was no statistically significant association between the presence of ALS and the levels of these oxysterols in the plasma of ALS patients [97].

#### 2.2.5. X-Linked Adrenoleukodystrophy

Adrenoleukodystrophy (ALD) is caused by an ABCD1 mutation, a genetic condition that often follows an X-linked inheritance pattern (X-ALD). The buildup of very long-chain fatty acids (VLCFA) is linked to this progressive neurodegenerative disorder. Severe cerebral inflammatory demyelination and milder spinal cord axonopathy are the primary manifestations of X-ALD [98]. The primary impacted areas of X-ALD are the adrenal cortex and inflammatory demyelinating lesions, where lipid peroxidation, particularly that generating oxysterols, primarily develops [99].

Patients with X-ALD have elevated levels of 7KC in their plasma. According to Nury, et al. [100], 7KC stimulates oxidative stress and peroxisomal dysfunction in microglial cells, which results in microglial cell activation and proliferation, which may be a factor in demyelination and dementia. By promoting brain inflammation through the activation of the NLRP3 inflammasome pathway, which is essential for demyelination and oligodendrocyte loss, 25-OHC was found to be a powerful mediator in the pathophysiology of X-ALD [101]. By triggering mitochondrial ROS, 25-OHC facilitates the assembly and activation of the NLRP3 inflammasome, which in turn causes oligodendrocyte death, IL-1β release, and microglia recruitment, ultimately resulting in severe neuroinflammation and demyelination [102].

#### 2.2.6. Autism Spectrum Disorder (ASD)

The hallmarks of autism spectrum disorder (ASD) include limited-repetitive patterns of behavior, interests, or hobbies, as well as ongoing deficiencies in social communication and engagement. It is now known that neuro-immune disorders and neuro-inflammation play a major role in the development and maintenance of ASD [103]. 24(S)-OHC was high in children and as a potential marker of ASD, although 7α-OHC and 25-OHC were only marginally significant. Age and 24(S)-OHC in patients had an inverse relationship [104]. Variants in the liver X receptor gene and oxysterol dysregulation in ASD were examined and it was found that 27-OHC may be used as an ASD diagnostic indicator [105]. While 27-R-OHC levels were lower in the ASD group than in the control group, 24-OHC and 25-OHC levels were significantly greater. The autistic group had a significantly greater ratio of 24(S)-OHC to 27-OHC. According to the receiver operating characteristic study, this ratio had “acceptable discrimination potential” and was statistically significant in its ability to distinguish between diagnoses of ASD and non-ASD [106].

### 2.3. Eye Diseases

The quality of life of millions of people is impacted by ocular degeneration, a significant public health concern, that includes cataracts, glaucoma, macular degeneration, and diabetic retinopathy. Oxysterols cause inflammation and cell death pathways and are linked to the pathogenesis of eye degeneration [107].

#### 2.3.1. Cataract

The most common cause of blindness globally is a cataract, which is defined as the loss of transparency in the natural lens of the eye [108]. Oxysterols could have a function in the formation of cataracts. They may change intracellular lipid homeostasis and Na^+^/K^+^ ATPase activity, which may be significant risk factors in cataract physiopathology [48]. Cataract development is linked to abnormalities in the metabolism of cholesterol, such as sterol 27-hydroxylase (CYP27A1) or 7-dehydrocholesterol reductase [109].

Studies conducted in clinics revealed that cataractous human lenses had higher levels of cholesterol, 7β-OHC, 7KC, 5α,6-α-epoxycholestanol, 20α-OHC, and 25-OHC than normal lenses [107].

Certain oxysterols, including 7β-OHC, 7KC, 5α, 6α-epoxycholestanol, 20α-OHC, and 25-OHC, were detected by gas chromatography in human cataracts retrieved after conventional eye surgery, but no cholesterol oxides were found in any healthy lens [110]. 7KC has also been detected in the lenses of cataract patients (around 4 nmol/mol of free cholesterol while it is undetectable in controls) [110,111].

#### 2.3.2. Age-Related Macular Degeneration (AMD)

In the retina, the macula is responsible for central vision and color perception. Disorders of the macula can be of genetic origin or are linked to age, such as AMD.

AMD and atherosclerosis may have comparable mechanisms. It is commonly recognized that oxysterols play a part in the development of atherosclerosis. Due to their cytotoxic, pro-inflammatory, and pro-oxidant qualities, oxysterols may be implicated in the retinal pigment epithelium and photoreceptor lesions that occur in AMD, since cholesterol is a component of drusens [112].

During AMD, oxidative stress appears in retinal pigment epithelial cells leading to the formation of oxysterols [26,113]. In primary cultures of pig retinal pigment epithelial cells, 24(S)-OHC, 25-OHC, or 7KC caused minor mitochondrial dysfunctions but a notable 2- to 4-fold increase in reactive oxygen species generation. Additionally, in decreasing order (25-OHC > 24(S)-OHC > 7KC), they increased IL-8 gene expression and IL-8 protein secretion [42].

Studies have shown a correlation between oxysterols and AMD, either at the level of the oxysterols or the genes that produce or bind them. In fact, substantial levels of 7KC have been discovered in the drusen, a type of proteolipidic deposit that is typical in cases of AMD [107].

7KC accumulates in the neural retina fractions of monkeys between 3 and 5 times more than in the pigment epithelium and choriocapillaris fractions. This accumulation occurs mainly at the level of drusen [114]. It has been shown that 7KC has chemoattractant activity towards retinal microglia, which triggers their activation and migration into the subretinal space, probably by a mechanism involving the inflammasome [115]. Additionally, on retinal pigment epithelial cells, 7KC induces the secretion of vascular endothelial growth factor (VEGF) [116].

Without any risk alleles in genes coding for complement factor H members, an allele in the cholesterol-24S-hydroxylase (CYP46A1) gene may increase the risk of exudative AMD [117].

### 2.4. Osteoporosis

Osteoporosis (OP), a systemic bone disease, is characterized by reduced bone strength, microarchitectural alterations, and an elevated risk of fracture. As they are involved in a number of critical biological processes, cholesterol oxidation products are significant substances in the preservation of bone metabolic equilibrium [118]. Certain research indicates that oxysterols such α-triol may have a role in osteoporosis [119]. Comparably, it was demonstrated that a major decrease in trabecular and cortical bone resulted from an increase in 27-OHC, either by injection or by genetically altering the CYP7B1 enzyme [120].

An endogenous selective estrogen receptor modulator (SERM), 27-OHC controls bone homeostasis by competitively binding to the estrogen receptor [121]. When 27-OHC concentration is raised pharmacologically, bone trabeculae and cortical bone are significantly reduced, which ultimately leads to OP [122].

22S-HOC and 20S-HOC, influence bone homeostasis by encouraging mesenchymal stem cells to differentiate osteogenically while preventing their lipogenic development [123]. 22R-OHC, 22S-OHC, and 20S-OHC have been shown to have pro-osteogenic effects on M2-B104 cells, whereas 7KC and cholestane-3beta-5alpha-6beta-triol have been shown to have anti-osteogenic effects on rat bone marrow stromal cells [119,124].

Using alveolar bone healing models and periodontal ligament stem cells, 22S-OHC and 20S-OHC together encourage periodontal regeneration [118,125].

According to Nelson, Wardell and McDonnell [121] and He and Nelson [126], 27-OHC interacts with the estrogen receptor and may also encourage osteoporosis [127]. Osteoblast differentiation is inhibited by 25-OHC [128]. The process of bone damage caused by exposure to combined metals (Fe and Pb) may involve 7KC. This could be connected to variations in inflammatory levels in vivo that promote osteoclast proliferation [129].

### 2.5. Sarcopenia

Sarcopenia is defined by a progressive loss of skeletal muscle mass and strength that occurs naturally with age [130]. Elevated amounts of pro-inflammatory cytokines like interleukin-6 (IL-6) and tumor necrosis factor-α (TNF-α), as well as C-reactive protein (CRP), are linked to sarcopenia. These pro-inflammatory cytokines change intercellular communication, raise adiposity, and disrupt insulin-like growth factor 1 (IGF-1) signalling in the skeletal muscles [131]. Skeletal muscle contraction can result in the production and release of cytokines known as myokines, such as apelin, which can impact skeletal muscle function and act on oxidative stress and inflammation [132]. It is likely that the positive effects of apelin on muscle metabolism are achieved by activating AMP-activated protein kinase and Akt, which in turn triggers mitochondriogenesis [133].

There was an increase in 7KC, and particularly 7β-OHC, in the plasma of sarcopenic patients [134]. The same authors established that the cytotoxic effects of 7β-OHC on myoblasts are somewhat greater than those of 7KC. These two oxysterols had less of an adverse effect on differentiated C2C12 cells (myotubes). Furthermore, apelin implicated in sarcopenia and inflammatory biomarkers (CRP, TNF-α, IL-6, IL-8, and LTB4) were also measured in the serum [135,136].

In their clinical investigation, Priyadarsini, Nanda, Devi and Mohapatra [130] verified that oxidative stress and inflammation are present in sarcopenic individuals and found new oxidative stress biomarkers, particularly oxysterols (7KC, 7β-OHC) produced by cholesterol autoxidation, that may influence the skeletal muscle atrophy.

Song, et al. [137] investigated how muscle-specific proliferator-activated receptor gamma coactivator-1 alpha (PGC-1α) regulates Nrf2 to modify mitochondrial oxidative stress in elderly sarcopenia. PGC-1α) and nuclear factor erythroid 2-related factor 2 (Nrf2) expression in C2C12 cells were decreased by either PGC-1α silencing or 7β-OHC treatment. Moreover, PGC-1α silence reduced the expression of the Nrf2 protein and elevated damaging ROS in the cells treated with 7β-OHC. Conversely, PGC-1α overexpression raised the production of the Nrf2 protein and reduced the damaging ROS in cells treated with 7β-OHC.

### 2.6. Bowel Diseases

Inflammatory diseases of the gastrointestinal tract include Crohn’s disease, ulcerative colitis, and inflammatory bowel diseases (IBDs). The first area of the body to be exposed to the effects of oxysterols is the gut, where the dietary sources of oxysterols, primarily foods high in cholesterol, come from. This main interaction may disrupt the balance of the human digestive system and contribute to injury to the intestinal mucosa [138].

Oxysterols could contribute to the evolution and worsening of these pathologies through their pro-inflammatory and pro-oxidant effects. Secretion of pro-inflammatory cytokines has been described on colon epithelial cells (Caco-2) treated with a mixture of 7KC, 5,6α-epoxycholesterol, 5,6β-epoxycholesteol, 7α-OHC, and 7β-OHC [139]. In inflammatory bowel diseases, oxysterols can participate in alteration of the intestinal barrier via activation of matrix metalloproteinases (MMP) which degrade cell junctions. Addition of a mixture of oxysterols composed of 7KC, 7α-OHC, 7β-OHC, and 5,6-epoxycholesterol to colonic epithelial cells induces their death by apoptosis [31,140]. The integrity of the intestinal epithelium and the vascular endothelium barrier can be weakened by 7KC and 25-OHC [141].

EBI2 (Epstein–Barr virus-Induced gene, or GPR183) is a G-protein coupled receptor that is activated by certain oxysterols, such as 7,25-diOHC [142]. In both steady state and during inflammation, production of colonic lymphoid structures is mediated via the EBI2-7,25-diOHC axis and enhanced oxysterol synthesis. Likewise, oxysterols encourage the downregulation of the CH25H enzyme, which may have multiple roles in the pathophysiology of intestinal fibrosis and inflammatory bowel disorders [143].

Independent of EBI2-mediated cell migration, a large intake of 25-OHC has been demonstrated to modify intestinal immunity, limit plasma cell differentiation, and impair IgA production and the response, through SREBP2 [144].

Inflammatory signals raise 7,25-diOHC, and during colitis, EBI2 regulated the recruitment of inflammatory cells. As a result, studies using EBI2-deficient mice show that in an innate model of intestinal inflammation, these animals were less prone to colitis. Increased synthesis of the EBI2 ligand 7,25-diOHC links colonic inflammation to the oxysterol EBI2 pathway. Furthermore, in patients with ulcerative colitis, there is a strong association between colonic inflammation and the expression of CH25H and CYP7B1 [145].

### 2.7. Lung Diseases

Three respiratory infections, tuberculosis (TB), severe acute respiratory syndrome-coronavirus-2 (SARS-CoV-2), and silicosis, have been demonstrated to raise the local synthesis of oxysterol in the lung [146].

#### 2.7.1. Tuberculosis

*Mycobacterium tuberculosis* (Mtb) is the causative agent of tuberculosis (TB), an infectious respiratory disease. A novel finding is that the host immunological response to Mtb infection is regulated by oxysterols and their receptors [146].

Compared to animals that were not infected, mice with Mtb infections had higher lung expression of the oxysterol-producing enzymes CH25H and CYP7B1 and increased levels of 25-OHC which correlated with this [147].

7α,25-OHC is the most powerful endogenous agonist, and both it and 25-OHC are ligands for the oxysterol-sensing receptor EBI2. Dendritic cells, eosinophils, macrophages, innate lymphoid cells (ILCs), T cells, and B cells are among the immune cells that express EBI2/GPR183 [146].

According to Bohrer, et al. [148], oxysterol synthesis triggers the migration of TB EBI2/GPR183^+^ immune cells to the lung, indicating the potential utility of EBI2/GPR183 and oxysterols as biomarkers for early TB diagnosis and prediction of disease severity. MTb catalyzes the change from 25-OHC to 25-hydroxycholest-4-en-3-one using the 3HSD enzyme. Consequently, MTb interferes with the human immune response by modulating the activity of 25-OHC through its enzymatic system [149]. However, people with chronic obstructive pulmonary disease, another illness linked to persistent infection, have higher than normal amounts of 25-OHC in their lungs [150].

In humans, the enzyme 3-hydroxysteroid dehydrogenase (3HSD) type 7 controls the amount of 7,25-dihydroxycholesterol. A 3HSD homologue found in MTb may functionally resemble 3HSD type 7 enzyme, obstructing immune cell movement driven by oxysterol [149].

When 7α,25-OHC activates EBI2/GPR183 in primary human monocytes, Mtb and *Mycobacterium bovis* BCG were restricted intracellularly. The addition of an EBI2/GPR183 antagonist eliminated this effect, indicating that this receptor regulates the intracellular growth of mycobacteria and maybe other microorganisms [151].

Investigation on THP-1-derived macrophages and primary human monocyte-derived macrophages showed that Mtb infection causes the production of IL-36, which in turn promotes synthesis of the LXR ligands 25-OHC and 27-OHC. The synthesis of antimicrobial peptides like cathelicidin and defensins, which improve mycobacterial control, is triggered by LXR activation [152].

#### 2.7.2. SARS-CoV-2 and Respiratory Diseases

In COVID-19 and influenza, there is evidence that oxysterols play a role in the immune response to severe viral respiratory infections [153]. Numerous investigations have demonstrated that oxysterol concentrations vary during SARS-CoV-2 infection. According to a study that tracked the kinetics of serum 25-OHC over time in a single female COVID-19 patient, 25-OHC levels significantly increased later in the infection when the patient’s clinical condition significantly deteriorated and peaked two days before the patient’s death [154]. In a different study, serum concentrations of 27-OHC were inversely correlated with disease severity, and 7KC and 7β-OHC were higher than in healthy matched controls [32].

In a different study of COVID-19 patients with various degrees of metabolic comorbidities there was a drop in 7KC and an increase in 25-OHC, 24(S)-OHC, and 27-OHC compared to healthy controls. The concentrations of 4β-OHC and 7α-OHC did not differ [155].

Single-cell sequencing of bronchoalveolar lavage samples from COVID-19 patients with moderate and severe disease revealed that the oxysterol-producing enzymes CH25H and CYP7B1 are increased in lung macrophages and myeloid dendritic cells, compared to those of healthy controls. Increased expression is associated with the severity of the disease. In addition, EBI2/GPR183 expression in macrophages increased with COVID-19 infection [152].

In a murine model of COVID-19 infection using a mouse-adapted strain of the virus, SARS-CoV-2 causes upregulation of CH25H and CYP7B1 in the lung, leading to increased synthesis of both 25-OHC and 7α,25-OHC [152]. In SARS-CoV-2 infection, the plasma level of 7KC and 7β-OHC are slightly but significantly increased [32]. 7KC is also increased in a serious inflammatory lung condition: silicosis [156].

The slowly progressing chronic respiratory disease known as chronic obstructive pulmonary disease (COPD) is distinguished by an obstructive ventilatory pattern [138]. In COPD, levels of ROS and oxysterols (7KC, 24(S)-OHC and 27-OHC) are increased [157]. Even at high levels of 27-OHC in their patients’ sputum, Kikuchi, et al. [158] showed an increase in the expression of CYP27A1 in the lung of COPD patients. Additionally, it has been shown that there is a negative correlation between lung function and the sputum level of 27-OHC. Through the activation of NF-kB and upregulation of TGF-1, 27-OHC is associated with the differentiation of lung fibroblasts into myofibroblasts and the synthesis of extracellular matrix protein [158]. The pathophysiology of COPD involves 27-OHC induced cellular senescence. In vitro, 27-OHC treatment of lung cells from COPD patients increased the expression of proteins linked to senescence. Furthermore, these patients have increased CYP27A1 expression in their alveolar macrophages and lung fibroblasts [159].

The progression of COPD has been linked to inducible bronchus-associated lymphoid tissue. The expression of CH25H and CYP7B1 in airway epithelial cells is upregulated in patients with COPD, which controls the establishment of inducible bronchus-associated lymphoid tissue in conjunction with B cell migration. Thus, the primary EBI2 ligand associated with inducible bronchus-related lymphoid tissue formation is 7,25-diOHC [160].

Pneumocytes and alveolar macrophages of COPD patients had elevated CH25H expression. Furthermore, sputum of COPD patients has higher levels of 25-OH, which is connected with neutrophil counts and sputum interleukin-8 levels [161].

Acute Lung Injury (ALI) is an acute systemic inflammatory process in lungs, which is clinically characterized by pulmonary infiltrates, hypoxemia, and edema [162].

In both of the ALI murine models examined, 25-OHC was the only oxysterol with altered levels during lung inflammation [163]. After intratracheal administration of LPS, most of the model’s characteristics, including leukocyte recruitment, mRNA expression, and inflammatory cytokine secretion, decreased whereas 25-OHC levels increased [163].

According to Zanjani, et al. [164], individuals with allergic asthma have higher plasma concentrations of 7KC, cholestane-3-,5-,6-triol, and malondialdehyde (MDA), a marker of oxidative stress. Leukocyte recruitment occurs in allergic inflammation, and this recruitment is reliant on the local synthesis of chemokines, cytokines, and other possible mediators. Thus, through a complicated signaling cascade including Ga_i_, cAMP, ERK, and Pin1, oxysterol–EBI2 signaling contributes to human asthma. Furthermore, even in patients with moderate asthma, Shen, et al. [165] showed a large spike in the count of eosinophils in their bronchoalveolar lavage, despite the patients having higher levels of 25-OHC, 7,27-diOHC, 27-OHC, and 7OHC. Increased numbers of inflammatory cells, including neutrophils, lymphocytes, and eosinophils, are associated with this elevated level of oxysterols.

#### 2.7.3. Silicosis

Silicosis is one of the chronic and irreversible occupational diseases. Crystalline silica dust, which has been connected to silicosis, is found in a variety of industrial settings, including pottery, ceramics, quarrying, and building. In addition to inflammatory respiratory conditions, patients with silicosis may also experience consequences like vasculitis, systemic sclerosis, and rheumatoid arthritis. Oxysterols and silicosis were found to be correlated. Patients with silicosis had considerably greater levels of plasma 7KC and C-triol than controls [156]. In silicosis, inflammation brought on by oxidative stress can lead to cholesterol auto-oxidation. The mean levels of 7KC in the silicosis patient and control groups were 40.61 ± 2.07 ng/mL and 20.26 ± 1.38 ng/mL, respectively [166].

### 2.8. Behcet’s Disease

Behçet’s disease (BD) is an acute systemic vasculitis which causes significant vascular damage and tissue ischemia at several body levels [167]. Oxysterols may play a role in the pathogenesis of BD, which is marked by immune system activation and significant changes in vascular wall cells, that result in vasculitis. Notably, patients with BD showed a raised carotid intima thickening and an atherogenic lipid level [168].

Through processes including cytotoxicity, oxidative stress, and inflammation, crucial for endothelial dysfunction, disruptions in the metabolism of cholesterol and lipoproteins which have been previously documented in BD, can affect vascular function [168,169].

Tunisian patients with BD have been shown to have abnormal levels of cholestane-3β,5α,6β-triol and 7KC in their plasma. There has also been a substantial reduction in 25-OHC levels in BD patients when compared to controls, but there has also been a significant increase in 27-OHC and cholestan-3β-ol (cholestanol) amounts in BD patients [170].

CH25H^−/−^ cells exhibited elevated inflammasome activity, resulting in an excess of IL-1β, a crucial proinflammatory cytokine in BD [171,172].

## 3. Mechanisms Associated with Oxysterol-Induced Inflammation

Numerous researchers have detailed the pathological effects of oxysterols, and various studies have linked them to a variety of inflammatory conditions, as described in Section 2. Research on the molecular pathways by which oxysterols influence the development and course of inflammation is an area of active investigation.

Since oxysterols are polyvalent structures, they have a variety of proinflammatory effects. Nevertheless, it is very challenging to investigate every pathway, and the molecular mechanisms by which oxysterols cause inflammation remain unclear. Numerous transcriptional regulators most likely control the expression of genes that are reliant on cholesterol [173].

Many chronic inflammatory diseases, including atherosclerosis, neurodegenerative disorders like Alzheimer’s and multiple sclerosis, dermatological diseases like psoriasis and bacterial skin infections, chronic neuropathic pain, adipose tissue inflammation linked to obesity, and non-alcoholic steatohepatitis (NASH) have been shown to have increased toll-like receptor (TLR) expression, activity, and ligand availability [174].

Herein we focus on the fact that exposure to oxysterols induces the activation of two distinct routes which result in proinflammatory phenomena. The first pathway is mediated by activating the TLR4 signaling complex and NF-kB signaling, whereas the second pathway includes activating liver X receptor (LXR) and reducing the amount of cholesterol in membranes [175].

### 3.1. Cytokinic/TLR4 Pathway

#### 3.1.1. Membrane Receptors

The most thoroughly researched and defined innate immune receptors are the TLR type I transmembrane receptors, of which 10 functional members have been identified in humans (TLR1 to TLR10). One of the most significant and extensively researched of these is TLR4 [175,176].

TLRs can be activated through the binding of a range of extrinsic and intrinsic ligands to their extracellular domain. This activation initiates receptor dimerization and subsequent binding of either the adaptor protein myeloid differentiation factor 88 (MyD88) or the Toll/IL-1R (TIR) domain-containing adaptor, that triggers the interferon-β (TRIF)-dependent pathway [177]. Ultimately, this cascade leads to the activation of interferon-regulating factor 3 (IRF3). Certainly, TLRs necessitate the activation of either the MyD88-dependent pathway (applicable to all TLRs except TLR3) or the TRIF-dependent pathway (pertinent to TLR4 and TLR3) to initiate subsequent signaling events [178].

This activation, in turn, triggers the activation of mitogen-activated protein kinases (MAPKs) and nuclear factor-kB (NF-kB) [179].

Subsequently, this cascade results in the release of various inflammatory molecules, thereby enhancing the local inflammatory response and/or contributing to matrix breakdown. Mounting evidence suggests the participation of TLRs, particularly TLR2 and TLR4, in the initiation, advancement, and vulnerability of various oxysterol-associated inflammatory diseases [178].

**Figure 2:**
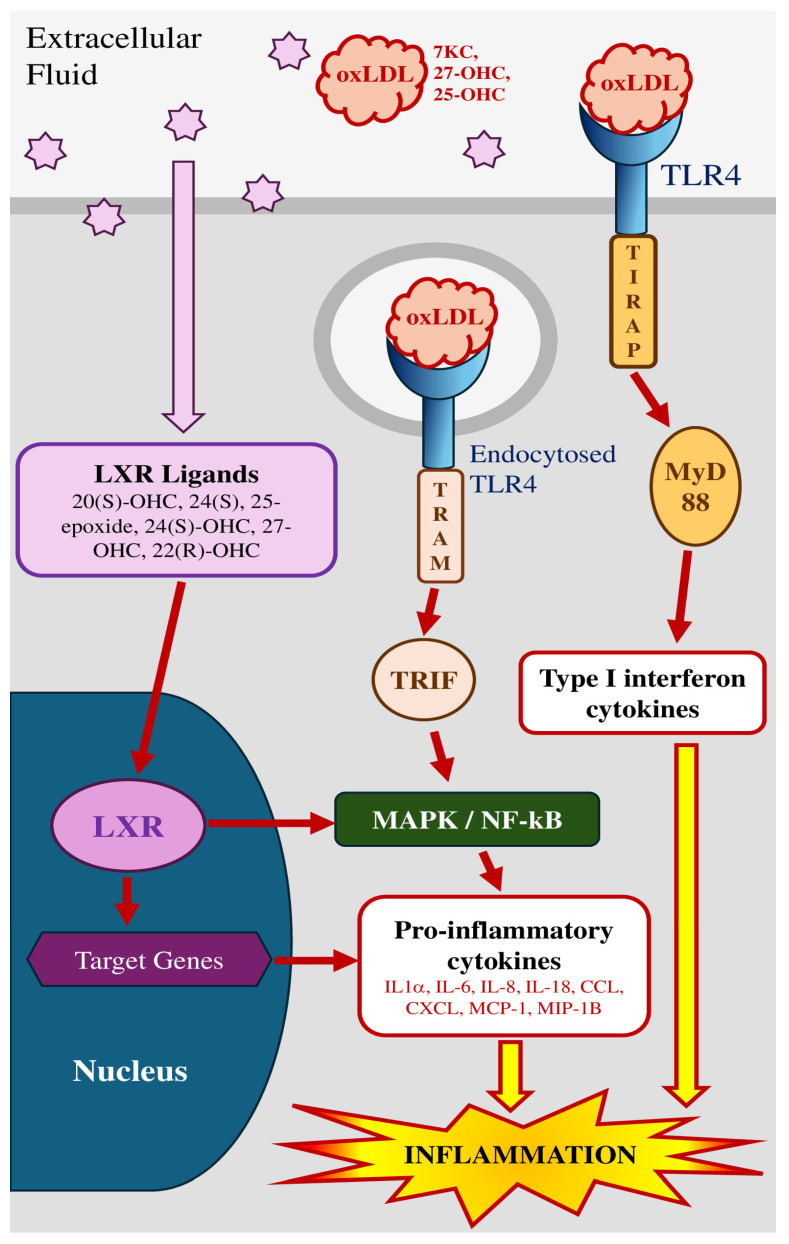
Oxysterol-induced cytokinic effects. Since they are fat-soluble, oxysterols can diffuse directly from the extracellular fluid, into the cytoplasm and across the nuclear envelope. Certain oxysterols are agonists on the nuclear LXRs, which can activate transcription of pro-inflammatory cytokines either directly, or via the MAPK and NF-kB pathways. Oxysterol components of oxLDL can also exert cytokinic effects through TLR4. At the cell-surface, oxysterols activate TLR4, recruiting toll-interleukin-1 (TIR) domain-containing adaptor protein (TIRAP) and myeloid differentiation primary response 88 (MyD88), stimulating the transcription of type 1 interferon cytokines. The binding of oxysterols also promotes dimerization and internalization of TLR4. Endocytosed TLR4 associates with TRIF-related adaptor molecule (TRAM) and TIR-domain-containing adaptor-inducing interferon-β (TRIF), enhancing the transcription of pro-inflammatory cytokines through the MAPK and NF-kB pathways.

In addition to the traditional exogenous ligands, endogenously generated TLR ligands include minimally modified low-density lipoproteins (LDLs) and oxidized LDLs. These ligands encompass active components such as oxidized phospholipids, oxysterols, and both free and esterified aldehydes [176]. For example, TLR4 is adept at identifying endogenous damage-associated molecular patterns (DAMPs), such as oxidized low-density lipoprotein (Ox-LDL), molecules affected by reactive oxygen species, and apoptotic cells. Upon engagement, TLR4 activates the production of inflammatory cytokines through co-receptors MD-2 and CD14, involving the signaling adaptors MyD88 and either MAL or TRIF [174] (Figure 2).

In addition, through a mechanism involving TLR2 and TLR4, it has been shown that components of oxLDLs, notably oxysterols, activate human and murine monocytes and macrophages during inflammatory processes. They also increase the expression of these receptors to promote and maintain the production of pro-inflammatory molecules [180].

Amongst oxysterols, 27-OHC plays a pivotal role in atherosclerosis pathogenesis, by activating diverse signal transduction pathways associated with immune and inflammatory responses, as well as oxidative stress [176].

In this theme, growing research indicates that exposure to oxysterols including 25-OHC and 7KC increases the synthesis of several inflammatory mediators in a range of cell types, such as IL-1α, IL-6, IL-8, monocyte chemoattractant protein-1 (MCP-1), and MIP-1β [175].

#### 3.1.2. Nuclear Receptors

Numerous cellular signaling pathways that affect cellular receptors, such as nuclear receptors, can be activated or inhibited by oxysterols. In their capacity as intracellular receptors, these proteins bind to lipophilic ligands that can pass through the plasma membrane. Members of the nuclear receptor superfamily that control cholesterol homeostasis include the liver X receptors (LXRs) [181].

LXRs are nuclear receptors that function as a security regulator to limit free cholesterol in tissues [182]. Numerous LXR target genes, such as ABCA1, ABCG1, SREBP-1c, and fatty acid synthase, are implicated in the metabolism of cholesterol and fatty acids. AIM/SPa is one of the other targets that regulates innate immune responses [181]. The physiologically principal endogenous LXR ligands are 20(S)-OHC, 24(S),25-EPOX, 24(S)-OHC, 27-OHC, and 22(R)-OHC [183,184]. LXRs are involved in the regulation of important metabolic pathways such as cholesterol homeostasis, glucose homeostasis, and lipogenesis in both humans and rats [185]. Oxysterol binding to LXRs regulates the protein expression of insulin-susceptible glucose transporter GLUT4 in fat tissue and muscle, thereby modulating glucose uptake. It has been demonstrated that LXRs may decrease inflammation through simulation-dependent and independent operations. It has been suggested that LXRs bind to NF-κB transcriptional complexes in an SUMOylation-dependent way to suppress the production of inflammatory genes in transcription. ABCA1 is an essential mediator of anti-inflammatory actions of LXRs [186].

Through the control of ABCG1 (cholesterol transporter) expression, LXR signaling links T-cell proliferation in the acquired immune response to cellular sterol metabolism. According to these studies, LXRs regulate a variety of inflammatory and immunological pathways [187].

As concerns pathway activating LXR, in their study, [188] discovered that in endothelial cells, oxysterol LXR agonists fully activate inflammatory pathways, which results in the down-regulation of eNOS and the up-regulation of adhesion molecules (ICAM1, VCAM1, SELE), chemokines (IL8, IL1α, CCLs and CXCLs), transcription factors (EGR1, FOS), and enzymes (COX2).

### 3.2. Non-Cytokinic Pathways: Leukotrienes, and Prostaglandins

Enzyme systems liberate and oxygenate arachidonic acid, which results in the production of eicosanoids, a significant class of inflammatory mediators. Eicosanoid release is now understood to be essential in the inflammatory process. For instance, the cyclo-oxygenase enzyme pathway produces prostaglandins and other prostanoids, which have strong inflammatory qualities [189].

Non-cytokine inflammation is characterized by the secretion of pro-inflammatory molecules such as arachidonic acid, prostaglandins, leukotrienes, thromboxanes, and prostacyclins [190].

Oxysterols can increase phospholipase A2 (PLA2) activity and arachidonic acid levels, which can lead to non-cytokine inflammation. Oxysterols enhanced the release of arachidonic acid and the formation of 11,15-dihydroxy-9-oxoprosta-5,13-dien-1-oic acid (PGE2) in normal rat kidneys that were activated by fetal calf serum [191]. At 24 and 48 h, 7KC dramatically increased the mRNA expression of COX-2 and 12-LOX, with both enzymes exhibiting considerably greater expression at 24 h compared to 48 h. Along with lipoxygenase and cyclooxygenase activities, prostaglandin E2 (PGE2) can also be elevated [192].

Panini, et al. [193] demonstrated that treatment with 25-OHC greatly boosted arachidonate release. Furthermore, an elevation in the concentration of intracellular Ca^2+^ activates cytosolic phospholipase A2 (cPLA2), causing the enzyme to relocate from the cytosol to the nuclear envelope/ER. Phosphorylation of Ser-505 by mitogen-activated protein kinases is the second mechanism by which cPLA2 is activated [193].

### 3.3. Contributions of Calcium and Potassium to Oxysterol-Induced Inflammation

#### 3.3.1. Calcium Channels

**Figure 3:**
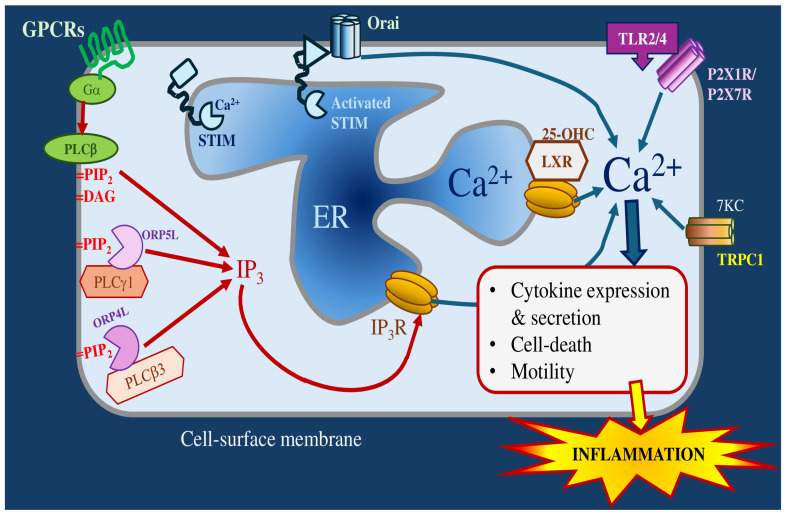
Pro-inflammatory effects of oxysterols through altered Ca^2+^ signalling. Certain oxysterols act as agonists for G-protein coupled receptors (GPCRs), including EBI2/GPR183. This activates phospholipase C (PLC) enzymes, through the alpha subunits of heterotrimeric G-proteins. PLC cleaves the membrane lipid phosphatidylinositol 4.5-bisphosphate (PIP_2_) into the second messengers diacylglycerol (DAG) and inositol-1,4,5-trisphosphate (IP_3_). The oxysterol-binding-protein-related proteins ORP4L and ORP5L also stimulate IP_3_ production, by recruiting PLC isozymes to PIP_2_ in the inner leaflet of the cell-surface membrane. IP_3_ is a cytoplasmic second messenger, which causes Ca^2+^ release from the endoplasmic reticulum (ER) by binding to IP_3_R/Ca^2+^ channels. On binding 25-OHC, LXRs can also stimulate IP_3_R Ca^2+^ release, via a non-genomic mechanism. Release of Ca^2+^ from the ER is sensed by stromal interaction molecules (STIM1 and 2), which migrate to sites of contact with the cell-surface membrane, where they activate Orai channels (Orai1, 2 and 3), in a process termed store-operated Ca^2+^ entry. By binding to TLR2 and TLR4, oxysterols can activate Ca^2+^ influx through the purinoreceptor channels P2X1R and P2X7R. Both oxLDL and 7KC can also stimulate Ca^2+^ entry through transient receptor potential, canonical type 1 (TRPC1) cation channels. Together, increases in Ca^2+^ via these mechanisms can promote inflammation, by enhancing the transcription and secretion of cytokines, stimulating the migration of pro-inflammatory cells, and triggering cell death, leading to the release of pro-inflammatory factors.

Ca^2+^ is a cytoplasmic second messenger that regulates most cellular processes, including gene expression, membrane transport, metabolism, motility, growth, and death [194]. Ca^2+^ also controls inflammation, modulating migration, chemotaxis, phagocytosis and transcription and secretion of cytokines [195] (Figure 3). The immune system is regulated by oxysterols, acting via a diverse range of ion channels and receptors [196]. In many cell types, certain oxysterol congeners influence cytoplasmic Ca^2+^ concentrations, but the molecular mechanisms underlying this signaling have not been fully resolved [197]. Consequently, it is anticipated that oxysterols can provoke inflammatory responses through Ca^2+^ signaling. The current review addresses ion transport mechanisms whose function is influenced by oxysterols and which play roles in inflammation.

The phospholipase C (PLC) family of enzymes cleaves the membrane lipid phosphatidylinositol-4,5-bisphosphate (PIP_2_) into the second messenger inositol-1,4,5-trisphosphate (IP_3_) and diacylglycerol. For most PLC isozymes, enzymatic activity is stimulated by the Ga_q/ll_ subunits of G-protein coupled receptors (PLC-b subtypes) or by receptor tyrosine kinases (PLC-g subtypes) [198]. IP_3_ binds to receptors (IP_3_Rs) located predominately in the ER, to open an intrinsic ion channel and release Ca^2+^ into the cytoplasm. PLCg subtypes can modulate TLR4 signaling by promoting endocytosis and by blocking the recruitment of the TIRAP adaptor protein [199]. In cystic fibrosis bronchial epithelial cells, PLC-b3 activation potentiates the pro-inflammatory release of IL8 stimulated by TLRs [200]. Even though it is coupled to Ga_i_ and not Ga_q/11_, the oxysterol-binding G-protein coupled receptor EBI2/GPR183 increases cytoplasmic Ca^2+^ in B-lymphocytes and in astrocytes [142,201].

TLRs modulate the PLC-IP_3_-IP_3_R signaling pathways. In rat cardiomyocytes, the pro-inflammatory mediator high-mobility group box 1 acts through TLR4 to enhance Ca^2+^ leak from the SR via oxidation of the RyR2 channel, thereby suppressing contractile function [202]. Similarly, in a rat model of sepsis, TLR4 activation by LPS in cardiomyocytes promotes mitochondrial ROS production, oxidation of RyR2 and SR Ca^2+^ leak [203]. In *Porphyromonas gingivalis* LPS-induced sepsis in mice, TLR4 caused cardiac dysfunction by activating NADPH oxidase-4, increasing ROS, stimulating calmodulin-dependent protein kinase-II with phosphophorylation of RyR2 and of phospholamban, a negative regulator of SR Ca^2+^ uptake [204].

In human macrophages, an oxysterol mixture increased levels of b1-integrin, a pro-inflammatory molecule involved in cell adhesion. This increase in b1-integrin required both Ga_q_ and PLC [205]. In a rat aortic smooth muscle cell line, incubation for 24 h with 7β-OHC, β-epoxide or 7KC all increased resting cytoplasmic Ca^2+^ concentrations. This treatment also decreased the abundance of two intracellular Ca^2+^-release channels, IP_3_R1 and the type 1 ryanodine receptor (RyR1) [206]. The anti-viral oxysterol, 25-OHC, is produced in an inflammatory response by immune cells. At the neuromuscular junction of the mouse diaphragm, sub-micromolar concentrations of 25-OHC suppressed neurotransmitter release, whereas levels above 1 mM enhanced neurotransmission. This potentiating effect required rapid non-genomic signaling through LXR, and IP_3_R-dependent Ca^2+^ release [95].

In many cell types, depletion of SR/ER Ca^2+^ stores by Ca^2+^ release channels and leak pathways is sensed by the stromal interaction molecules 1 (STIM1) and 2 (STIM2). Subsequently, these proteins translocate to junctions between the ER and cell-surface membrane, where they open Orai Ca^2+^-influx channels. This process is termed store-operated calcium entry (SOCE) [207] and potentially acts as a nexus between inflammation and oxysterol-mediated signaling. Oxysterols exert their effects on SOCE by binding to oxysterol binding protein-related proteins (ORPs). ORP-5 and -8 bind to PIP_2_, increase mitochondrial Ca^2+^ and enhance cell proliferation [208]. In T-lymphocytes, ORP5L recruits PLC-g1 to membrane regions enriched in PIP_2_ to facilitate IP_3_ production and Ca^2+^ release [209]. Also in T-lymphocytes, ORP4L promotes translocation of PLC-b3 from the nucleus to the plasma membrane [210], where it activates subplamalemmal IP_3_R1 channels [211]. 25OHC is produced by macrophages during inflammation and increases intracellular Ca^2+^ in diaphragm skeletal muscle, a tissue with abundant macrophages and which is continually exposed to oxidative stress. These increases in Ca^2+^ suppress contraction and involve release from intracellular Ca^2+^ stores, but does not require RyRs, LXRs, or EBI/GPR183. However, 25OHC can act as a selective estrogen receptor modulator and an estrogen receptor antagonist blocks diaphragm Ca^2+^ increases caused by this oxysterol [212].

TLRs also modulate SOCE in the regulation of inflammatory processes. In human mesenchymal stem cells, the TLR3 agonist polyinosinic-polycytidylic acid increases the abundance of IP_3_R3, Orai2, Orai3 and STIM1, promotes SOCE and leads to increased cytokine secretion [213]. BV2 microglial cells contain Orai1 and STIM1, with SOCE being activated by TLR agonists. A SOCE inhibitor (CM-EX-137) reduced Ca^2+^ influx, iNOS activation, and suppressed TLR-dependent inflammatory transcription through NFATc and NFkB [214]. Stimulation of human endothelial progenitor cells with LPS activated TLR4 to promote exosome release, through a mechanism requiring IP_3_Rs and SOCE [215]. In mouse spinal astrocytes, Orai1 is required for full TLR4 activated cytokine production [216]. In breast cancer cells, the SOCE antagonist BTP2 suppressed migration, proliferation, and the production of inflammatory cytokines [217]. In contrast, LPS attenuated SOCE, downregulated Orai1 and STIM1, and upregulated TLR4 in the satellite glial cells of rat sympathetic ganglia [218].

In contrast, the suppression of TLR3 stimulated release anti-viral cytokines in human airway epithelial cells by extracellular nucleotides or histamine utilized Ga_q_, but did not involve SOCE [219].

The transient receptor potential (TRP) superfamily are cation channels which are gated by a variety of stimuli, including changes in membrane potential, temperature, membrane lipids, acidic pH, and noxious substances. Although inflammatory stimuli, particularly those involving TLR4, can provoke Ca^2+^ influx via a wide range of TRP channels [220,221], the reported effects of oxysterols are more restricted. In THP1 monocytes, 7KC induces apoptosis by recruiting the TRP canonical type 1 (TRPC1) to lipid rafts and promoting Ca^2+^ influx [222]. TRPC1 also activates oxLDL-stimulated Ca^2+^-dependent apoptosis of vascular smooth muscle cells [223].

The ionotropic purine receptors P2X7R and P2X1R are known to be gated by TLR4 and TLR2, respectively [224,225]. In human pigmented retinal epithelial cells, oxysterols activate P2X7R causing cell death. In the case of 25-OHC, this required recruitment of pannexin-1 (a protein that forms large membrane pores), but cell death was pannexin-independent for 7KC [226]. In human keratinocytes, 25-OHC caused P2X7R-dependent pyroptosis [227].

#### 3.3.2. Potassium Channel

In addition to Ca^2+^ transporters, K^+^ channels play key roles in inflammatory processes, by modifying membrane potential (and consequently the gating of voltage-dependent ion channels), regulating cell volume and controlling cell motility [228]. In mouse 158 N oligodendrocytes and in BV2 microglia, 7KC and 24(S)-OHC increased the abundance of the voltage-gated potassium channel K_v_3.1b, leading to elevated cytoplasmic K^+^ levels and cell death [229]. At low micromolar concentrations, side-chain modified oxysterols (20S-OHC, 22R-OHC, 24(S)-OHC, 25-OHC, 27-OHC) inhibited opening of the large conductance Ca^2+^-dependent K^+^ channel, Slo1 [230]. In intestinal epithelial cells, TLR2, TLR4 and TLR7 activation decrease K^+^ conductance, whereas TLR5 increases it. The Ca^2+^-dependent K_Ca_3.1 and the inwardly rectifying K_ir_6 are key sites of TLR4 action, and were proposed as targets for therapeutic approaches the treatment of inflammatory bowel disease [231].

## 4. Prevention of Oxysterol-Induced Inflammation

In an effort to regulate inflammatory and/or chronic immunological processes, a number of researchers are attempting to modify the immune response that is triggered in human immune cells by dietary and endogenous cholesterol oxidation products.

Despite the ineffectiveness of TLR4 antagonists, like eritoran, to successfully prevent acute sepsis, the discovery of TLR4 modulators as prospective therapeutics for chronic inflammatory disorders whose pathophysiology appears to involve TLR4 remains an active area [174].

On the other side, diseases where oxysterols may be involved can be avoided, diminished, or treated using natural or synthetic molecules, as well as by combinations of such molecules. Vitamins, fatty acids, phospholipids, terpenes, phenols, plant pigments, antioxidants, oils, and plant extracts are among these molecules. Synthetic molecules such as memantine and simvastatine are also included, as well as dimethyl fumarate (DMF) and its main metabolite, monomethylfumarate (MMF) (Table 1) [232].

### 4.1. Natural Molecules

Numerous studies have used a variety of models mimicking pathophysiological settings to examine the ability of dietary phytochemicals to protect against oxysterol toxicity.

#### 4.1.1. Tocopherols

Tocopherols are fat-soluble substances, including vitamin E. They comprise group of eight molecules, including four tocopherols (α-, β-, γ-, and δ-tocopherol) and four tocotrienols (α-, β-, γ-, and δ-tocotrienol) [296].

α-tocopherol is an antioxidant that is a strong suppressor of the generation of superoxide anions that occurred during treatment with 7KC and prevented subsequent apoptosis [44,88,265]. Likewise, several studies have demonstrated that α-tocopherol has a cytoprotective impact on numerous cell types such as human promonocytic leukemia cells (U937) treated with 7KC [233]. Furthermore, the effects of α-tocopherol on murine microglial BV-2 cells was examined by Debbabi, Nury, Zarrouk, Mekahli, Bezine, Sghaier, Grégoire, Martine, Durand, and Camus [57]. These authors demonstrated that α- and γ-tocopherol could inhibit the loss of mitochondrial transmembrane potential (ΔΨm) caused by 7KC. In 7KC-induced oxiapoptophagy, downregulation of the GSK3/Mcl-1, Nrf2, and PDK1/Akt signaling pathways was concurrently noted and α-tocopherol stopped the occurrence of these events [239].

α-tocopherol significantly reduced all cytotoxic effects caused by 7β-OHC in on murine C2C12 myoblasts, which is why this bioactive substance is so valuable in preventing peroxisomal damage (pexotherapy) [296]. When combined with DHA, α-tocopherol has a higher cytoprotective effect on 7β-OHC than when taken separately [88]. The toxicity caused by 24(S)-OHC is also prevented by α-tocopherol and DHA [297].

At least one explanation for α-tocopherol’s cytoprotective function is its capacity to stop oxysterol from building up in lipid rafts, which inhibits the activation of signals that cause cell death [240].

#### 4.1.2. Carotenoids and Other Terpenoids

Carotenoids are terpene group of compounds that are created when 5-carbon isoprene molecules bond together. They have light yellow to dark red color and dissolve in chemical solvents and oils [296].

Astaxanthin, lutein, canthaxanthin, and β-carotene all prevented the production of 7KC caused by 2,20-azobis-isobutyronitrile (AIBN) [242]. The oxidative stress, apoptosis, and pro-inflammatory cytokine cascade in human THP-1 macrophages were inhibited by co-incubating lycopene (2 μM) with 7KC (15–25 μM) [243]. This was achieved by reducing ROS formation by NADPH-oxidase (NOX–4), blocking redox-sensitive MAP kinases, increasing PPARγ levels (which has anti-inflammatory properties), and blocking pro-inflammatory NF-κB transcription factor. In another study, lycopene inhibited the rise in pro-inflammatory cytokine expression and secretion caused by oxysterols (7KC and 25-OHC). This resulted in a reduction of NF-κB activation, p38 mitogen-activated protein kinase (MAPK) phosphorylation, and ROS generation produced by oxysterols. The NF-κB inhibitor pyrrolidine dithiocarbamate similarly imitated the suppression of cytokine activation mediated by oxysterol. Furthermore, in THP-1 macrophages, the carotenoid raised the levels of receptor γ, activated by peroxisome proliferator [244]. However, in human monocytic U937 cells, 7β-OHC (30 μM)-induced apoptosis and downregulated Akt/PKB activity, which could not be stopped by lycopene or astaxanthin (0.1–1 μM) [235].

Through TLR4 reduction and improved ABCA1 mediated clearance, co-treatment of SH-SY5Y cells with bornyl acetate (BA) and menthol (ME) being significantly decreased lipid accumulation and amyloid production caused by 7KC. In these cells, co-treatment with BA and ME concurrently reduced intracellular calcification, mitochondrial damage, acetylcholinesterase activity, and oxidative stress due to 7KC. Additionally, cells exposed to 7KC exhibit higher amounts of misfolded protein indicators and apoptotic mediator mRNA, whereas cells cotreated with BA and ME showed considerably lower levels of these markers. Moreover, co-treatment of 7KC-induced cells with BA and ME reduced the protein production of amyloidogenic, proinflammatory, and proapoptotic markers [245].

Phytosterols are phytonutrients which resemble cholesterol oxidation products in both structure and activity. There are two types of phytosterols: 24 methylsterols (campesterol) and 24 ethylsterols (sitosterol and stigmasterol) [2].

In rat glioma cells treated with 7KC, spinasterol decreased mitochondrial activity (30–50%), while schottenol decreased 50% mitochondrial activity in 158 N cellsand 10–20% in C6 cells [246].

#### 4.1.3. Phenolic Compounds

Phenolic chemicals are naturally occurring metabolites defined by an aromatic ring with one or more hydroxyl substituents. Polyphenols are divided into flavonoids such as flavonols, flavones, flavan-3-ols, anthocyanidins, flavanones, and isoflavones; and non-flavonoids such as stilbenes and phenolic acids [298].

Numerous phenolic substances, such as phenolic acids, tannins and flavonoids have cytoprotective properties against oxysterols. The majority of these substances are found in large amounts in the Mediterranean diet. For example, resveratrol, at 1 μM, displayed cytoprotective action against 7KC (5–40 μM) in human retinal ARPE-19 cells. Therefore, compared to cells treated with 7KC alone, the total number of viable cells in ARPE-19 cells treated with 7KC and this phenolic acid was significantly higher, and the amount of vascular endothelial growth factor (VEGF) secreted by 7KC was significantly decreased [251].

Resveratrol can also skew the M1 (pro-inflammatory)/M2 (anti-inflammatory) balance towards an anti-inflammatory profile by inhibiting the upregulation of several pro-inflammatory and proangiogenic molecules induced by 7KC (15 μM) in human monocytes isolated from peripheral blood [252]. The toxicity caused by 7KC (50 μM; 48 h) in mouse neuronal N2a cells was greatly decreased when it was combined with resveratrol, quercetin and apigenin (3.125 and 6.25 μM). The reduction of ROS production in whole cells and prevention mitochondrial dysfunction were noticed [239]. Trans-resveratrol, quercetin, and apigenin are strong inhibitors of 7KC-induced oxiapoptophagy [276].

Monocyte–endothelial cell adhesion mediated by 7KC is inhibited by epigallocatechin 3-gallate (EGCG) [250]. The protective effect of epigallocatechin-3-gallate at 1 mM against the pro-inflammatory effect of an oxysterol mixture (30 mM) was also demonstrated on human CaCo-2 cells. This was achieved by caspase-3 activation, reduction of pro-inflammatory and chemotactic genes (IL-8, IL-1a, IL-6, IL-23, MCP-1, TGF-b-1, TLR2 and TLR9), and overexpression of the ROS-generating NADPH oxidase isoform NOX1 [249].

The polyphenols found in Sardinian wine may also have an indirect effect by completely inhibiting the pro-oxidant and pro-inflammatory processes that dietary oxysterols cause by obstructing oxysterol-related NOX1 activation [258]. In the same way, according to Testa, Gamba, Badilli, Gargiulo, Maina, Guina, Calfapietra, Biasi, Cavalli, and Poli [52], pretreatment with quercetin (5 mM for 1 h) loaded in b-cyclodextrin nanoparticles prevented the neuro-inflammatory activity exerted by 24(S)-OHC, 27-OHC, and 7b-OHC alone (5 mM), or as a mixture (15 mM), in human neuroblastoma SH-SY5Y cells through the TLR4/cyclooxygenase-2/membrane bound prostaglandin E synthase signaling cascade. Similarly, wine phenolics can down-modulate the signaling pathway involving NOX1 and p38 MAPK activation, which is activated by oxysterols in intestinal cells, thereby reducing NF-κB activity in intestinal inflammation [259].

The phenolic alcohols of olive oil, hydroxytyrosol and tyrosol, along with their sulfate metabolites, were found to prevent the pro-oxidative activity of an oxysterol combination that contained 7KC and 7β-OHC [254]. Through the modification of p38 and JNK pathways, extra virgin olive oil polyphenols, hydroxytyrosol, tyrosol, and homovanillic alcohol significantly inhibited the production of reactive oxygen species (ROS) and MAPK phosphorylation in human peripheral blood mononuclear cells. These effects were observed in response to oxysterol-induced cytokine secretion [260]. The same authors demonstrated that pretreatment with the olive oil phenolic extract counteracted the effects of oxysterols on the human colon adenocarcinoma cell line (Caco-2). This was achieved, at least in part, by modulating the MAPK-NF-kB pathway. This extract directly modulates p38 and JNK1/2 phosphorylation and activation of NF-kB [261]. Theobromine also inhibits oxysterol-induced cell damage in Caco-2 cells. Theobromine is found in dark chocolate and cocoa bean shell extracts with varying polyphenol levels [139,257].

#### 4.1.4. Betalains

Betalains are naturally occurring pigments that dissolve in water and are found in large quantities in plants including red and yellow beets, amaranth, prickly pears, pitaya, and others. Red-violet betacyanin and yellow betaxanthin are two examples of betalains. For ages, they have been used extensively as food coloring additives [299].

These compounds impacted significantly on the negative outcomes of oxysterols. In this line, indicaxanthine, which is a betalain isolated from cactus pear, inhibits apoptosis caused by 7KC in human monocytic THP-1 cells [262]. Indicaxanthine also prevents eryptosis, the depletion of glutathione (GSH), the release of PGE2, and the influx of Ca^2+^ in healthy human erythrocytes caused by a mixture of oxysterols (7KC, 7α-OHC, 7β-OH, cholestan-3β, 5α,6β-triol, 5α,6α-epoxycholesterol, and 5β,6β-epoxycholesterol) for 48 h [263].

### 4.2. Plant Extracts

Plant extracts demonstrate cytoprotective effects against oxysterols. These include methanolic extract of *Clinacanthus nutans* [266], ethanolic mint leaf extracts [265], red yeast rice extract (Xuezhikang) [264], ethanol-water extract from *Carpobrotus edulis* [267], and anthocyanin-rich Aronox extract from *Aronia melanocarpa* E that inhibits apoptosis, ROS generation and the decrease in the transmembrane mitochondrial potential induced by 7β-OHC [269]. In addition, by altering Krüppel-Like Factor 2 and adhesion molecules in 7KC-treated HUVECs, soy leaf extract exhibits atheroprotective properties [268].

*Digera muricata*, which is rich in sitosterol, was also used to modulate the NF-KB/iNOS signaling pathway in macrophages to counteract atherogenic reactions mediated by lipopolysaccharide and 7KC. Treatment with *D. muricata* resulted in decreased gene expression of proatherogenic mediators (iNOS, COX-2, MMP9, IL-6, IL-1β, CD36, and CD163) and increased gene expression of anti-atherogenic mediators (MRC1 and PPARγ) in macrophages [270].

### 4.3. Edible Oils and Fatty Acids

#### 4.3.1. Edible Oils

Vegetable oils including olive, argan oils, and milk thistle seed oil that are prevalent in the Mediterranean diet are considered to be cytoprotective molecules against the deleterious effects of oxysterols. Using 158 N and BV-2 cells Badreddine, Zarrouk, Karym, Debbabi, Nury, Meddeb, Sghaier, Bezine, Vejux, and Martine [238] and Meddeb, Rezig, Zarrouk, Nury, Vejux, Prost, Bretillon, Mejri, and Lizard [272] demonstrated that a 7KC-induced cell death mechanism, could be prevented by argan, olive, and milk thistle seed oil. Argan oils and milk thistle seed oil decreased plasma membrane permeability to propidium iodide (PI) in 158 N rat oligodendrocytes and inhibited 7KC (25 μM)-induced ROS overproduction [238]. Olive and argan oils can stop the loss of plasma membrane esterase activity caused by 7KC (25 μM) in 158 N and BV-2 cells. This activity is assessed by staining with fluorescein diacetate, which is also employed as a criteria for cell death [300,301].

Regarding Nigella seed oil, Meddeb, Rezig, Zarrouk, Nury, Vejux, Prost, Bretillon, Mejri, and Lizard [272] found that while it does not exhibit any cytoprotective impact against 7KC on 158 N cells, it does shield C2C12 murine myoblasts from the oxysterol-induced death of cells.

Maaloul, Ghzaiel, Mahmoudi, Mighri, Pires, Vejux, Martine, de Barros, Prost-Camus, and Boughalleb [274] studied the effects of *Silybum marianum* (SMSO), *Silybum eburneum* (SESO), and *Silybum marianum* commercial (SMCSO) seed oils on 7KC and 7β-OHC induced oxidative ROS overproduction in THP-1 cells. SMCSO significantly reduced the oxidative damage of 7KC on THP-1 (by 30.38%), with this being the most effective method, according to the study. SMSO also showed a significant diminution of 27.87% and 22.51%, respectively. However, SESO only resulted in a non-significant reduction of 19.27%.

Sea urchin egg oil normalizes the fatty acid profile and lessens cell death, which are negative effects caused by 7β-OHC. Additionally, this oil reduces the synthesis of MDA and CD and regulates the activity of antioxidant enzymes [273].

Numerous compounds present in these oils, such as α-tocopherol, fatty acids and polyphenols, have been demonstrated to dramatically reduce 7KC- and 7β-OHC-induced cytotoxicity, and may be responsible for these cytoprotective properties [297].

#### 4.3.2. Fatty Acids

Vegetable oils include several kinds of fatty acids for instance ω3, ω6, and ω9 including α-linoleic acid (C18:2 n-6); eicosapentaenoic acid (C20:5 n-3); doco-sahexaenoic acid (DHA; C22:6 n-3); oleic acid (C18:1 n-9); and sporadic acid (a C19 cyclopropene fatty acid). These fatty acids are effective in preventing 7KC- and 7β-OHC-induced cytotoxicity in human retinal epithelial ARPE-19 cells and murine and human nerve cells (158 N, BV-2, N2-a, and SK-N-BE) [297,300]. Oleic acid has a protective impact on 7KC-induced mitochondrial and peroxisomal dysfunction in mouse BV2 microglial cells [271]. When 7KC was esterified with oleic acid no negative effects are seen in human monocytic U937 cells [278]. Elaidic acid was shown to increase plasma membrane fluidity in murine microglial BV2 cells; and oleic acid was able to stop the rise in plasma membrane fluidity caused by 7KC [271].

The fatty acids could operate via lowering oxidative stress and mitochondrial malfunction that results in cell death, as well as neutralizing 7KC by esterification [240].

Important changes in glutathione peroxidase, superoxide dismutase, and catalase activities, as well as in the amount and expression of glutathione peroxidase-1, superoxide dismutase-1, and catalase, were linked to decreased oxidative stress in 7KC-induced oxiapoptophagy. α-linolenic acid, eicosapentaenoic acid, docosahexaenoic acid, and oleic acid, also counteracted these effects. DHA regulates the expression of the Gpx4 gene and activates the glutathione and thioredoxin antioxidant systems in murine hippocampus HT22 cells for neuroprotection [241].

Fatty acids such as ω-3 and ω-9 fatty acids (α-linolenic acid (C18:3n-3), eicosapentaenoic acid (C20:5n-3), docosahexaenoic acid (C22:6n-3), erucic acid (C22:1n-9) and oleic acid (C18:1 n-9)), and Lorenzo’s oil (a 4:1 mixture of oleic and erucic acid) were tested for their cytoprotective effects using 158 N oligodendrocytes [240].

It is hypothesized that the plasma membrane modification seen with MC540 and in the presence of 7KC may be, at least partially, the result of lipid peroxidation as the same FAs (ALA, EPA, DHA, and OA) decreases the 7KC-induced ROS overproduction shown with DHE on both 158 N and ARPE-19 cells [240].

Short chain fatty acids (SCFAs) inhibit the toxic effects of 7KC and 7β-OHC on retinal epithelial cells. Sterculic acid prevents choroidal neovascularization and counteracts inflammation caused by 7KC [277].

Sterculic acid antagonizes 7 KC-mediated inflammation and inhibits choroidal neovascularization, and decreases the formation and the activation of Nlrp3 inflammasome induced by 7KC [279].

In murine macrophagic RAW264.7 cells, phospholipid bis(monoacylglycero)phosphate (BMP) inhibits the production of 7KC [280].

### 4.4. Probiotics and Microbial Enzymes

Probiotics are live bacteria that enhance or replenish the gut microbiota, which has been connected to better health. Probiotics are seen to be the contemporary equivalent of a panacea, with claims that they may treat or prevent a variety of illnesses in both adults and children [302].

Casula, Pisano, Serreli, Zodio, Melis, Corona, Costabile, Cosentino, and Deiana [284] discovered the probiotics *Lactiplantibacillus* plantarum 299v and *L. casei* DG’s capacity to protect intestinal cells from dietary oxysterol pro-oxidant and pro-inflammatory effects. These probiotics reduced the alteration of intestinal epithelial Caco-2 cell monolayer permeability caused by oxysterols. They were implicated in the regulation of ZO-1, JAM-A, occludin, and tight junction (TJ) proteins in connection to redox-sensitive MAPK p38 activation.

Butyrate, a member of a family of gut microbial metabolites, prevents choroidal neovascularization and lessens the development and activation of the 7KC-induced Nlrp3 inflammasome [279].

A possible strategy to address the deficiency of 7KC catabolism is medical bioremediation, which relies on the employment of microbial enzymes to make up for lost catabolic capabilities. Schloendorn [303] reported 7KC degradation by a *Pseudomonas aeruginosa*. Degradation from an initial value of 7KC (1000 ppm) was 88%. One potential enzyme implicated in the breakdown process is cholesterol oxidase [283]. *Rhodococcus erythropolis* MTCC 3591 is also identified as a potential degrader strain of 7KC. Under optimized conditions, this strain is able to degrade 93% of an initial concentration of 1 g/L 7KC [282].

*Thermobifida fusca* IP1 was evaluated for its capacity to break down 7KC in an M9 liquid media and demonstrated that it used 7KC as its only source of carbon and energy. When 7KC was tested in liquid culture, it was completely degraded and after twelve days it had completely disappeared from the sample [285].

### 4.5. Synthetic Molecules

#### 4.5.1. Monomethyl Fumarate and Dimethyl Fumarate

Among the synthesized compounds that have been demonstrated to be effective in decreasing 7KC toxicity in 158 N cells monomethylfumarate (MMF), the primary metabolite of dimethyl fumarate (DMF), and AG126, a tyrosine kinase inhibitor [297]. DMF is thought to work by activating the Nrf2 pathway. In 158 N murine oligodendrocytes cells, DMF attenuates 7KC-induced apoptosis, 7KC -induced LC3-I conversion to LC3-II and reduces 7KC -induced overproduction of O_2_^•−^ and H_2_O_2_ [288]. In 158 N cells, 7β-OHC-induced cytotoxicity is significantly reduced by DMF and its primary metabolite MMF [289]. In 158 N cells incubated with 7KC or 7β-OHC, the cytoprotective effect of dimethylfumarate (DMF; Tecfidera) and biotin (vitamin B8), which are used for the treatment of MS, have been shown [289].

#### 4.5.2. UDP-003

A brand-new family of cyclodextrin (CD) molecules was created to encapsulate harmful oxidized fats. To create CDs that eliminate atherogenic oxidized cholesterol from cells and tissues, a synergistic rational drug design approach combining in vitro, ex vivo, and in silico techniques is employed. A resulting molecule, UDP-003, has a great safety record and selectively binds and removes 7KC from both in-vivo and ex-vivo systems [304]. In mouse and human monocyte and macrophage cell lines, Bhargava, et al. [305] clarified the molecular processes of UDP-003 in reducing the deleterious effects of 7KC. In RAW 264.7 cells, UDP-003 inhibits and reverses the buildup of intracellular lipids. UDP-003 successfully removed 7KC from human vascular tissue. They also showed that administering UDP-003 can rejuvenate foam cells by restoring phagocytic function and reducing the buildup of intracellular lipid droplets and reactive oxygen species (ROS).

#### 4.5.3. Sulfo-N-Succinimidyl Oleate

Sulfo-N-succinimidyl oleate (SSO) is a synthetic derivative of oleic acid, which is cytoprotective against 7KC-induced cell death in ARPE-19 epithelial retinal cells. SSO did not exhibit any harmful effects throughout a broad concentration range. It is noteworthy that the cytoprotective effects of SSO on 7KC-induced oxiapoptophagy, a caspase-dependent mechanism of cell death in 158 N cells, were not linked to a buildup of lipid droplets. Furthermore, it inhibited 7KC-induced oxidative stress and cell death in ARPE-19 cells [240]. SSO also attenuates the toxicity of 7KC in 158 N oligodendrocytes, strongly reducing plasma membrane disorganization, oxidative stress, mitochondrial and peroxisomal dysfunction, as well as oxiapoptophagy [297]. In retinal epithelial ARPE19 cells, these different compounds also strongly reduced 7KC-induced oxidative stress, mitochondrial dysfunction, ROS overproduction, and cell death [251]. Noteworthy, among the different compounds studied, SSO as well as α-tocopherol (used as reference cytoprotective molecule) were the only compounds that could prevent the toxicity of 7KC without causing simultaneous accumulation of lipid droplets [240]. According to these authors, the SSO acts on mitochondrial interaction proteins (Mcl-1, Bad) to activate signaling pathways that restore ΔΨm and stop cell death. In addition, SSO is also able to protect against 24(S)-OHC- and Triol-induced cell death considered to play key roles in age-related diseases. SSO opposes peroxisomal metabolic dysfunction, a property which is not currently described for DMF, MMF or UDP-003.

#### 4.5.4. Other Molecules

Although their effects against 7KC toxicity have not yet been assessed, other compounds have been shown to be cytoprotective in a variety of cell types, including monocytes, endothelial cells, and nerve cells. These include mangafodipir (a contrast agent used in liver magnetic resonance imaging) [306], dimethylsulfoxide (DMSO), which prevents 7β-OHC-induced apoptosis in U937 cells by preserving lysosomes and mitochondria [306], and mitochondrial permeability transition pore (MPTP) inhibitors (ADP analogues; but not carboxya-tractyloside) [307].

In vitro, oxysterol-induced inflammatory activation can be inhibited and the endothelium barrier restored by the non-vitamin K antagonist (VKA) oral anticoagulant rivaroxaban. Rivaroxaban reduced the mRNA expression of IL-33, TNF-α, chemokines MCP-1, ICAM-1, VEGF, and tissue factor in HUVECs pre-stimulated with oxysterol [292].

Simvastatine, an inhibitor of the hydroxymethylglutaryl-CoA reductase (HMG-CoA) significantly attenuates 7KC-induced apoptosis in ARPE-19 cells as evidenced by decreased caspase 3/7 activity [232].

Certain molecules have activity against 7β-OHC, but not against 7KC. This is the situation with Trolox, a hydrophilic counterpart of vitamin E that is 3,4-dihydro-6-hydroxy-2,5,7,8-tetramethyl-2H-1-benzopyran-2-carboxylic acid [247]

Oxy210 and related analogs have anti-inflammatory effects on macrophages exposed with lipopolysaccharide in vitro. These effects are achieved through suppression of toll-like receptor 4 (TLR4), TLR2, and AP-1 signaling. The suppression of macrophage polarization is associated with Oxy210’s anti-inflammatory properties [174].

Azelnidipine, a calcium channel blocker, inhibits ROS-dependent expression of vascular cell adhesion molecule 1 (VCAM-1) induced by 7KC [293].

K-80003, a non-steroidal anti-inflammatory medication, is a retinoid X receptor α (RXRα) modulator that prevents 7KC-induced RXRα cytoplasmic translocation [294].

A novel regulator, 3β-sulfate-5-4 cholestenoic acid (3SCA), of cholestenoic acid (CA), 25-OHC, and 27-HOC metabolism and inflammation was discovered. 3SCA inhibited the gene expression of pro-inflammatory cytokines produced by LPS in human macrophages and markedly decreased the expression of genes involved in their metabolism in hepatocytes [295].

Fingolimod (2-amino-2-[2-(4-octylphenyl)ethyl]propane-1,3-diol; glatiramer acetate; FTY720; used at 30 and 500 nM) did not demonstrate any cytoprotective effects against oxysterols in evaluated in 158 N and N2a cells [297]. Ebselen, an organo-selenium medicinal molecule recognized for its antioxidant qualities, has not been shown to have also any cytoprotective benefits against 7β-OHC-induced cell death [247].

## 5. Conclusions

Extensive research has been performed on a number of oxysterol-related topics. It is clear that oxysterols have a significant impact on human diseases and inflammation. However, less is known about how these compounds affect the different organs and disorders associated with them. It is also extremely difficult to examine each pathway, and it is still unknown how exactly oxysterols produce inflammation at the molecular level. Studies are undoubtedly challenging due to the multitude of oxysterol forms, routes, and cell types involved. It is worthwhile to investigating the inflammatory activity of oxysterol pathways in many organs.

It is also worth generating functional foods or medications that will effectively reduce and/or cure inflammatory diseases where the levels of oxysterols are elevated, since there is an extensive list of natural (tocopherols, terpenoids, polyphenols, betalains, plant extracts, vegetable oils and fatty acids, probiotics, and microbial enzymes), and synthetic molecules including MMF, DMF, UDP-003, and SSO that prevent oxysterol-induced cytotoxicity and inflammation.

In this direction, the study of Kopp [308] comes to the conclusion that although aging raises the risk of non-communicable diseases, lifestyle factors are important because they may provide opportunities for prevention and intervention. Attempting to restore the physiological balance, for example, by implementing dietary changes (such as the Mediterranean, Okinawan, or Paleolithic diets) in conjunction with a combination of pharmaceutical therapies and other lifestyle adjustments, is one potential strategy.

Despite, the possible therapeutic potential of these compounds on human inflammatory diseases related to oxysterols, it is recommended that more research should be conducted to comprehensively determine their mechanism of action. In addition, it is likely that more substances with cytoprotective effects against oxysterols will be discovered and that mixtures of such substances might have synergistic effects.

## Figures and Tables

**Table 1:** Summarizing the experimental evidence related to natural and synthetic molecules that affect oxysterol toxicity.

Compounds Family	Nature Compounds (Concentration)	Used Cells	Oxysterols (Concentration)	References
Natural compounds				
*Tocopherols*	*α-tocopherol (100 mM)	Human pro-monocytic cells (U937)	7KC (40 µg/mL)	[233]
	*α-tocopherol (100 mM)	U937	7β-OHC and 7KC (200, 50, and 100 mM)	[234]
	*α-tocopherol (10 μM)	U937	7β-OH (30 mM)	[235]
	*α, γ-tocopherols (100 μM)	Rat aortic smooth muscle cells (A7r5)	7KC (20 μg/mL)	[236]
	*α-tocopherol (400 μM)	Murine oligodendrocytes cells (158 N)	7KC (12.5, 25, 50, and 100 μM)	[237]
	*α-tocopherol (400 μM)	158 N	7KC, 7β-OHC and 24S-OHC	[88]
	*α, γ-tocopherols (400 μM)	Murine microglial cells (BV-2)	7KC (50 μM)	[57]
	*α-tocopherol (400 μM)	158 N	7KC (25–50 μM)	[238]
	*α-tocopherol (400 μM)	Murine neuronal cells (N2a)	7KC (50 µM)	[239]
	*α-tocopherol (400 μM)	158 N and human retinal epithelial cells (ARPE-19)	7KC (50 μM, 100 μM)	[240]
	*α-tocopherol (400 μM	N2a	7KC (50 μM)	[241]
*Terpenes*	*β-carotene and oxygenated carotenoids lutein, canthaxanthin, astaxanthin (0.25–1 mM), and Lycopene (0.5–2 μM)	human THP-1 macrophages	7KC (4–25 μM) and 25-OHC (2–4 μM)	[242,243,244]
	*Lycopene and astaxanthin (0.1, 0.5 and 1 mM)	U937	7β-OHC (30 mM)	[235]
	*Bornyl acetate and menthol (50–1000 μg/mL)	SH-SY5Y, a human neuroblastoma cell line	7KC (50–100 μg/mL)	[245]
	*Spinasterol and schottenol (5, 10, 20, and 40 μM).	158 N, C6 rat glioma cells, and SK-N-BE human neuronal cells	7 KC (25–50 µM)	[246]
*Polyphenols*	*Resveratrol (10 µM))	U937	7β-OH and cholesterol-5β, 6β-epoxide (30 μM)	[247]
	*Epicatechin (5–10 μM)	Murine Monocyte/Macrophage (J774A,1)	7KC (20 μM)	[248]
	Apigenin (0.5, 2, 5, 10, 50 mM)	U937	7β-OH (30 mM)	[235]
	*Epigallocatechin-3-gallate (1 μM)	human colonic epithelial cells (CaCo-2)	Oxysterol mixture (30 μM)	[249]
	*Epigallocatechin-3- gallate (30–50 μM)	Human angiosarcoma cells (ISO-HAS)	7KC (50 μM)	[250]
	*Resveratrol (1.5–30 μM)	N2a, human monocytes, ARPE-19	7KC (15–150 μM)	[251,252]
	*Resveratrol, apigenin and quercetin (1.5–25 µM)	N2a	7KC (50 μM)	[239]
	*Hydroxytyrosol and tyrosol (2.5–10 μM)	CaCo-2	7KC (1875 μM)	[253,254]
	*Homovanillic alcohol (5–25 μM)	CaCo-2	7KC (25–50)	[253,255]
	*Taxifoline (dihydroquercetin) (15 μM)	Rat pheochromocytoma cells (PC12); SH-SY5Y	7KC (125 μM)	
	*Cocoa bean shell extracts with different polyphenol content (10 μg/mL, 25 μg/mL or 50 μg/mL)	Differentiated CaCo-2	dietary oxysterol mixture (Oxysterol mixture; 60 μM).	[256]
	*Theobromine (10 μM)	CaCo-2	Mixture of dietary oxysterols (60 μM)	[257]
	*Polyphenols extracted from wine (25 μg/mL) or caffeic acid, gallic acid, catechin and epicatechin (10 μM), quercetin (1 μM)	Differentiated CaCo-2-	Oxysterols-mixture (30 and 60 μM)	[258]
	*Quercetin or quercetin loaded into nanoparticles (5 µM)	SH-SY5Y	24-OHC, 27-OHC, and 7β-OHC (5 µM)	[52]
	*Phenolic compounds extracted from wines (25 μg/mL) caffeic acid, gallic acid acid, (+)-catechin, and (−)-epicatechin (10 μM), quercetin (1 μM)	Differentiated CaCo-2	Oxysterols-mixture (60 μM)	[259]
	*Olive oil phenolics: hydroxytyrosol, tyrosol, and homovanillic alcohol (0.25, 0.5, 1 μM)	Peripheral blood mononuclear cells (PBMCs)	Oxysterols mixture (20 μM)	[260]
	*Olive oil polyphenols (1–25 μg/mL)	CaCo-2	Oxysterols mixture (60 μM)	[261]
*Betalains*	*Indicaxanthin (1–5 μM)	THP-1 andhuman erythrocytes	7KC (7–16 μM)	[262,263]
*Plant extracts*	*Red yeast rice extract (100 μg/mL)	RAW 264.7	7KC (70 μM)	[264]
	*Ethanolic mint leaf extract (25–400 μg/mL)	RAW 264.7	7KC (20 μM)	[265]
	*Clinacanthus nutans (Lindau) extract (100 μg/mL)	hCMEC/D3 human brain endothelial cells	7KC (30 μM)	[266]
	*Carpobrotus edulis extract (20–200 µg/mL)	158 N	7β-OHC (20 μg/mL)	[267]
	*Soy-leaf ethanolic extract (100 μg/mL)	Human umbilical vein endothelial cells (HUVECs)	7 KC (20 µg/mL)	[268]
	*Anthocyanin-rich extract from *Aronia melanocarpa* E (50 μg/mL)	HUVECs	7β-OH (20 μg/mL).	[269]
	*Digera muricata extract (25, 50, 100, 200, 400, 600, 800 and 1000 µg/mL)	IC-21 macrophage cell line	7KC (2, 4, 6, 8, 10 µg/mL)	[270]
*Edible oils*	*Olive oil (1/1000)	158 N	7 KC (50 μM)	[57,271]
	*Argan and olive oils (1/1000, 1/1000)	158 N	7 KC (50 μM)	[238]
	*Milk thistle seed oil (3/1000)	158 N	7 KC (50 μM)	[272]
	*Sea urchin egg oil (10–600 μg/mL)	158 N	7β-OHC (50 μM)	[273]
	*Silybum seeds oil (60 mg/mL)	THP-1	7KC and 7β-OHC (25–50 µM)	[274]
*Fatty acids*	*Docosahexaenoic acid (50 μM)	158 N	7KC, 7β-OHC and 24S-OHC (25–50 µM)	[88]
	*Docosahexaenoic acid (50 μM)	SK-N-BE	7KC, 7α and 7β-OHC, 6α and 6β–OHC, 4 α and 4β-OHC 24S-OHC and 27-OHC (50–100 μM)	[275]
	*Oleic acid (200 μM)	BV-2	7KC (50 μM)	[57]
	*Oleic acid and elaidic acid (50, 100, 200, 300, 600 μM), docosahexaenoic acid (12, 25, 50 and 100 μM)	BV-2	7KC (25–50 μM)	[271]
	*α-linolenic, eicosapentaenoic and docosahexaenoic acids (50 μM)	N2a	7 KC (50 μM)	[276]
	*Docosahexaenoic acid (50 μM)	BV-2, 158 N	7 KC (25–50 μM)	[88]
	*Sterculic acid (1 μM)	ARPE-19	7KC (12 μM)	[277]
	Hydroxycholesteryl-3-oleate (50 μ M), 7-ketocholesteryl-3-oleate (100 μM)	U937	7 β-OHC (50 μM), 7KC (100 μM)	[278]
	*Acetate, propionate and butyrate	HUVECs	7KC	[279]
	*Bis(monoacylglycero)phosphate	RAW264.7	7KC	[280]
	*α-linolenic acid, eicosapentaenoic acid, docosahexaenoic acid, erucic acid, and oleic acid (100 μM), Lorenzo’s oil (a mixture of oleic and erucic acid, 4:1)	158 N and ARPE-19 cells	7KC (50 μM and 100 μM)	[240]
	*α-linolenic acid, eicosapentaenoic acid, docosahexaenoic acid, oleic acid (1.5 to 200 μM)	N2a	7KC (50 μM)	[241]
Probiotics and microbial enzymes	- Cholesterol oxydase (*Chromobacterium* DS-1) - bacteria *Rhodococcus erythropolis* MTCC 3951	Human fibroblasts	7KC (25–50 μM)	[281,282]
	*Pseudomonas aeruginosa* PseA	Bacterial culture	7KC (1 g/L)	[283]
	*Lactiplantibacillus plantarum* and *Lacticaseibacillus casei* (70 μL/mL)	CaCo-2	Oxysterols-mixture (100 μM)	[284]
	*Thermobifidafusca IP1*	Bacterial culture	7KC (0.0025 M)	[285]
Synthetic molecules				
	*N-acetyl-l-cysteine (10–15 mM)	U937, murine osteoblats (MC3T3- E1)	KC (100 μM)	[233,286]
	*Aminothiols glutathione (10–15 mM)	U937	7KC (100 μM)	[233]
	*Ergothioneine (0.1–1 mM)	Human brain endothelial cells (hCMEC/D3)	7KC (30 μM)	[287]
	*Dimethyl fumarate/Monomethylfumarate (25–50 μM)	PC12	7KC (100 μM)	[232,288]
	*Biotin (10 and 100 nM)	158 N	7β-OHC (50 µM)	[289]
	*UDP-003 (25, 50, 100, and 150 μM)	RAW 264.7	7KC (10 μM, 40 μM)	[290]
	*Sulfo-N-succinimidyl oleate (25–50 µM)	158 N and ARPE-19 cells	7KC	[240]
	*Memantine (1 μM, 1 mM)	158 N	7KC (25–50 μM)	[232]
	*Simvastatine (0.01 μM, 0.05 μM)	ARPE-19	7KC (75 μM)	[232]
	*Mangafodipir (15 μM to 800 μM)	U-937	7β-OHC (25 μM)	[291]
	*Rivaroxaban (100 and 500 ng/mL)	HUVECs	25-OHC (10 μg/mL)	[292]
	*Trolox (10 mM) ebselen (2 mM/L)	U937	7β-OH and cholesterol-5β, 6β-epoxide (30 µM)	[247]
	*Azelnidipine (0.1 μM)	U937	7KC (20 μM)	[293]
	*K-80003 (20 μM)	RAW264.7 Bone marrow cells	7KC (40 μM)	[294]
	*3β-Sulfate-5-4 cholestenoic acid	Primary human hepatocytes, Huh-7, and HepG-2 cells	Cholestenoic acid, 25-OHC, 27-OHC (20 μM)	[295]

## Data Availability

No new data were created or analyzed in this study. Data sharing is not applicable to this article.
